# Untargeted Metabolomics Profiling of Arabidopsis WT, *lbr-2-2* and *bak1-4* Mutants Following Treatment with Two LPS Chemotypes

**DOI:** 10.3390/metabo12050379

**Published:** 2022-04-22

**Authors:** Benedict C. Offor, Msizi I. Mhlongo, Paul A. Steenkamp, Ian A. Dubery, Lizelle A. Piater

**Affiliations:** Department of Biochemistry, University of Johannesburg, Auckland Park, Johannesburg 2006, South Africa; benedictoffor@gmail.com (B.C.O.); mmhlongo@uj.ac.za (M.I.M.); psteenkamp@uj.ac.za (P.A.S.); idubery@uj.ac.za (I.A.D.)

**Keywords:** *Arabidopsis thaliana*, BAK1, untargeted metabolomics, LBR2, LPS, *Pseudomonas syringae*, *Xanthomonas campestris*

## Abstract

Plants perceive pathogenic threats from the environment that have evaded preformed barriers through pattern recognition receptors (PRRs) that recognise microbe-associated molecular patterns (MAMPs). The perception of and triggered defence to lipopolysaccharides (LPSs) as a MAMP is well-studied in mammals, but little is known in plants, including the PRR(s). Understanding LPS-induced secondary metabolites and perturbed metabolic pathways in Arabidopsis will be key to generating disease-resistant plants and improving global plant crop yield. Recently, Arabidopsis LPS-binding protein (LBP) and bactericidal/permeability-increasing protein (BPI)-related proteins (LBP/BPI related-1) and (LBP/BPI related-2) were shown to perceive LPS from *Pseudomonas aeruginosa* and trigger defence responses. In turn, brassinosteroid insensitive 1 (BRI1)-associated receptor kinase 1 (BAK1) is a well-established co-receptor for several defence-related PRRs in plants. Due to the lack of knowledge pertaining to LPS perception in plants and given the involvement of the afore-mentioned proteins in MAMPs recognition, in this study, Arabidopsis wild type (WT) and mutant (*lbr2-2* and *bak1-4*) plants were pressure-infiltrated with LPSs purified from *Pseudomonas syringae* pv. *tomato* DC3000 (*Pst*) and *Xanthomonas campestris* pv. *campestris* 8004 (*Xcc*). Metabolites were extracted from the leaves at four time points over a 24 h period and analysed by UHPLC-MS, generating distinct metabolite profiles. Data analysed using unsupervised and supervised multivariate data analysis (MVDA) tools generated results that reflected time- and treatment-related variations after both LPS chemotypes treatments. Forty-five significant metabolites were putatively annotated and belong to the following groups: glucosinolates, hydroxycinnamic acid derivatives, flavonoids, lignans, lipids, oxylipins, arabidopsides and phytohormones, while metabolic pathway analysis (MetPA) showed enrichment of flavone and flavanol biosynthesis, phenylpropanoid biosynthesis, alpha-linolenic acid metabolism and glucosinolate biosynthesis. Distinct metabolite accumulations depended on the LPS chemotype and the genetic background of the *lbr2-2* and *bak1-4* mutants. This study highlights the role of LPSs in the reprogramming Arabidopsis metabolism into a defensive state, and the possible role of LBR and BAK1 proteins in LPSs perception and thus plant defence against pathogenic bacteria.

## 1. Introduction

Lipopolysaccharides (LPSs) as microbe-associated molecular patterns (MAMPs) from Gram-negative bacteria has been shown to trigger innate immune responses in animals and plants [1,2]. While LPS perception in mammals is well-studied, little is known about the role(s) of this MAMP and/or its epitopes and defence signalling in plants. In addition, it is yet to be resolved if the unknown LPS receptor(s) require co-receptors or accessory proteins. Sanabria et al. [3] proposed the involvement of a LPS-responsive *N. tabacum* S-domain receptor-like kinase (RLK) (*Nt-Sd-RLK*) gene encoding conserved B-lectin, S- and Plasminogen/Apple/Nematode (PAN) domains in LPS perception, while Arabidopsis AtLBR-1 and AtLBR-2 have been shown to perceive LPS and trigger defence responses such as upregulation of the *PR1* gene [4]. Using transcriptomics, Arabidopsis AtLBR-2 was further reported to play a key role in defence responses against LPSs [5]. Ranf et al. [6] reported that LPSs from *Xanthomonas* and *Pseudomonas* were sensed by the bulb-type (B-type) lectin S-domain (SD)-1 RLK (lipooligosaccharide-specific reduced elicitation, LORE) and induced a defence response in Arabidopsis. This finding was later set aside as a consequence of a subsequent result that LORE did not perceive LPS, but rather bacterial 3-hydroxy fatty acids co-purified with LPS [7].

The plant metabolome is often referred to as the terminal downstream product of the central dogma of biology and a bridge that links the genotype and phenotype [8]. Metabolomics evaluates small molecules that are synthesised or transformed as a result of a cellular metabolism [8,9]. As such, this ‘omics’ approach can complement the use of other ‘omics’ technologies since metabolites may not be directly encoded by the genome, and also changes in the transcriptome or proteome do not always correlate with the phenotypes [10]. Untargeted metabolomics has been used to profile LPS-induced defence-related metabolites such as glucosinolates, flavonoids, hydroxycinnamic acid (HCA) derivatives and lipids in Arabidopsis [11,12]. Moreover, Mareya et al. [13] using liquid chromatography coupled to mass spectrometry (LC-MS), reported defence-related metabolome changes that involved alterations in the levels of phytohormones and several other metabolites in *Sorghum bicolor* cells treated by the LPS from *Burkholderia andropogonis*.

The current study reports on purified LPSs from *Pseudomonas syringae* pv. *tomato* DC3000 (*Pst*) (LPS*_Pst_*) and *Xanthomonas campestris* pv. *campestris* 8004 (*Xcc*) (LPS*_Xcc_*) as MAMPs to treat Arabidopsis wild type (WT) and mutants (*lbr2-2* and *bak1-4*) generated by T-DNA insertional mutagenesis. The two LPS chemotypes differ in the chemical ‘molecular patterns’ of the LPS sub-moieties [11,14,15,16]. Both LPSs from *Pst* and *Xcc* have intact O-polysaccharides that characterises a smooth LPS which may differ in molecular structures. In addition, most phytopathogenic bacterial species have been shown to possess the [α-L-Rha-(1 → 3)-α-L-Rha-(1 → 3)-α-L-Rha-(1 → 2)]*_n_* repeating motif that has been linked to induction of defence responses [17]. The acylation pattern of lipid A moiety contributes to the variability of LPS and affects its endotoxicity [18]. While the lipid A moiety of LPS from *Xcc* is hexa-acylated, that of *Pst* is either penta-/hexa-acylated [16,19,20]. With regards to the mutant lines, LBP/BPI-related proteins (LBRs) were shown to perceive LPS from bacterial *Pseudomonas aeruginosa* and trigger defence responses [4]. In turn, brassinosteroid insensitive 1 (BRI1)-associated receptor kinase 1 (BAK1) is a well-established co-receptor for several defence-related PRRs in plants [21,22,23].

Understanding the LPS-induced metabolites and perturbed metabolic pathways in Arabidopsis WT and mutants (*lbr2-2* and *bak1-4*) will reveal the role of this MAMP, specifically pertaining to two chemotypes, as well as LBR and BAK1 proteins in plant defence against Gram-negative bacterial pathogens. To investigate LPS-induced metabolome changes in the mutant lines vs. the WT, an untargeted metabolomics approach using LC-MS was followed and chemometric models were used for the data analyses. The observed dynamic cellular metabolomic variations due to the different LPS chemotypes treatments thus contribute to efforts in elucidating molecular and biochemical mechanisms underlying LPS perception in plants and could be extrapolated to plant: microbe interactions. In addition, the results obtained in this study will contribute to future research in identifying biomarkers in crops for resistance to diseases, and as such, improve crop yield.

## 2. Results

LPS has been shown to cause innate immune responses in animals and plants. While the LPS recognition and defence signalling is well-documented in mammals, little is known about its role in plants, including a postulated PRR responsible for ligand perception. In this study, a knockout (KO) of Arabidopsis LBR2 was selected because it has been shown to recognise LPS from bacterial *P. aeruginosa*, while BAK1 has been established as an essential co-receptor to several known PRRs such as flagellin-sensitive 2 (FLS2), elongation factor-thermo unstable (EF-Tu) receptor (EFR), etc. As such, an untargeted metabolomics approach was followed to study the role of different LPS chemotypes from *P. syringae* (*Pst*) and *X. campestris* (*Xcc*) in the metabolome dynamism of Arabidopsis WT and mutants (*lbr-2-2* and *bak1-4*). From here on, LPS from *Pst* and *Xcc* are abbreviated as LPS*_Pst_* and LPS*_Xcc_*, respectively, while early LPS treatments represent 0–12 h time points and later treatments represent 18–24 h.

### 2.1. Ultra-High Performance Liquid-Chromatography-Mass Spectometry Data Analysis

The hydromethanolic leaf extracts from Arabidopsis WT and mutant (*lbr2-2* and *bak1-4*) plants treated with LPS*_Pst_* and LPS*_Xcc_* were prepared from tissue harvested after 0, 12, 18, and 24 h post-treatments. These samples were analysed using a UHPLC-qTOF-MS instrument, in both electrospray ionisation (ESI) modes (+/−). The instrument generated base peak intensity (BPI) MS chromatograms that depicted well-resolved peaks, with time- and treatment-related peak differences, distributed over 0–30 min, in Arabidopsis WT and mutant plants treated with both LPS chemotypes. Figure S1 shows representative WT ESI (−) data. When compared to the controls, these observed treatment- and time-related peak differences/intensities suggested the underlying Arabidopsis metabolome changes that may arise from the use of different LPS chemotype treatments (i.e., differences in response to LPS chemotypes by WT vs. mutant plants).

### 2.2. Multivariate Data Analysis

To further evaluate the effect of LPS*_Pst_* and LPS*_Xcc_* on the metabolomes of Arabidopsis WT and mutant (*lbr2-2* and *bak1-4*) plants, as observed from the differential chromatographic profiles of the respective leaf extracts, and to extract valuable biological information from the multidimensional data, chemometric and multivariate data analysis (MVDA) tools were used. The data was processed using MarkerlynxXS^TM^ and exported to ‘Soft independent modelling of class analogy’ (SIMCA) software for principal component analysis (PCA), hierarchical clustering analysis (HiCA), orthogonal projection to latent structures discriminant analysis (OPLS-DA) and OPLS-DA loading S-plots modelling. The unsupervised PCA modelling is vital for the analysis of multivariate data due to its ability to reduce the dimensionality of data, and reveal natural groupings and trends within and between the data sets [24]. The PCA models were validated using the cumulative modelled variation that explains the variation (R^2^) and the models’ predictive ability (Q^2^). In addition, HiCA dendrograms were used to support treatment-related sample grouping observed in PCA models. Here, PCA scores plots and HiCA dendrograms revealed time-related grouping of samples treated with LPS*_Pst_* and LPS*_Xcc_* in the Arabidopsis WT, *lbr2-2* and *bak1-4* plants. Representative WT ESI (−) data is shown in Figure S2. The differential PCA and HiCA dendrograms sample clustering in WT and mutants supported evidence of metabolic reprogramming of the Arabidopsis metabolome subsequent to different LPS chemotype treatments.

**Figure 1:**
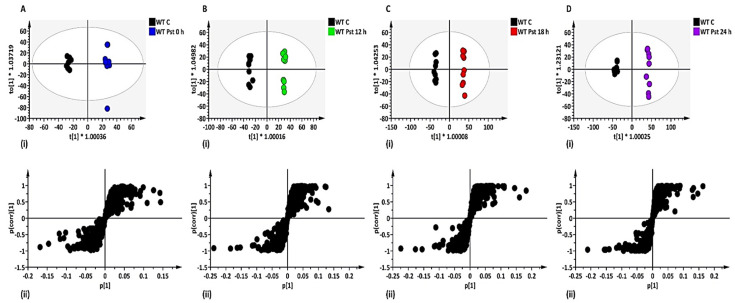
Orthogonal projection to latent structures discriminant analysis (OPLS-DA) modelling of Arabidopsis WT leaf extracts (ESI (−) data). (**A**–**D**) (**i**) represent OPLS-DA score plots showing clear separation between control vs. LPS*_Pst_* treatment after 0, 12, 18, and 24 h, respectively. (**A**–**D**) (**ii**) represent OPLS-DA loading S-plots showing the discriminant features (ions) responsible for the sample grouping observed in (**A**–**D**) (**i**). The OPLS-DA model parameters were: (**A**) R^2^X = 46.2%/Q^2^ = 97.4%, (**B**) R^2^X = 53.6%/Q^2^ = 98.3%, (**C**) R^2^X = 60.9%/Q^2^ = 98.9%, (**D**) R^2^X = 64.2%/Q^2^ = 98.9%, respectively. The variables in the top right quadrants of the S-plots correlated positively to the treatment. Selected discriminant ions for downstream metabolite identifications were based of a correlation [*p*(corr)] of ≥ 0.5 and covariance of (p1) ≥ 0.05. The equivalent set of figures for the ESI (+) mode is presented as Figure S3.

**Figure 2:**
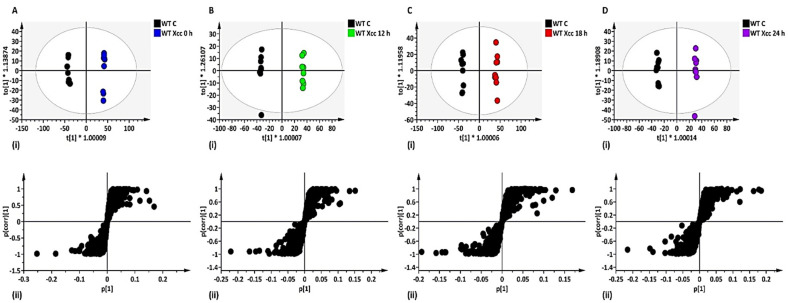
OPLS-DA modelling of Arabidopsis WT leaf extracts, (ESI (−) data). (**A–D**) (**i**) represent OPLS-DA score plots showing clear separation between control vs. LPS*_Xcc_* treatment after 0, 12, 18, and 24 h, respectively. (**A–D**) (**ii**) represent OPLS-DA loading S-plots showing the discriminant features (ions) responsible for the sample grouping observed in (**A–D)** (**i**). The OPLS-DA model parameters were: (**A**) R^2^X = 57.8%/Q^2^ = 99.3%, (**B**) R^2^X = 48.9%/Q^2^ = 99.0%, (**C**) R^2^X = 64.0%/Q^2^ = 99.3%, **(D)** R^2^X = 45.5%/Q^2^ = 98.5%, respectively. The variables in the top right quadrants of the S-plots correlated positively to the treatment. Selected discriminant ions for downstream metabolite identifications were based on a correlation [*p*(corr)] of ≥ 0.5 and covariance of (p1) ≥ 0.05. The equivalent set of figures for the ESI (+) mode is presented as Figure S4.

To evaluate the cause of sample grouping observed in PCA, supervised modelling such as OPLS-DA score plots were subsequently used. Additionally, OPLS-DA loading S-plots were included to highlight and extract LPS-induced metabolite ‘features’ (ions with a specific Rt and *m/z*), contributing to the group separation observed in OPLS-DA score plots [25]. For example, the Arabidopsis WT OPLS-DA models showed clear separation of control vs. 0, 12, 18 or 24 h treated with LPS*_Pst_* (Figure 1A–D for ESI (−) and Figure S3A–D for ESI (+)), as well as the LPS*_Xcc_* (Figure 2A–D for ESI (−) and Figure S4A–D for ESI (+)). These models were validated, and an example of receiver operator characteristic (ROC) plots of control vs. 0, 12, 18, and 24 h post-LPS*_Pst_*-treated Arabidopsis WT are shown in Figure S5. ROC plots summarised the performance of OPLS-DA as an excellent binary classifier with 100% sensitivity and 100% specificity. Overall, differential separations observed in OPLS-DA models, when comparing control vs. treated samples in both WT and mutants (*lbr2-2* and *bak1-4*), can be linked to changes in the metabolome that may arise from different LPS chemotype treatments. Subsequently, the LPS-induced significant variables of OPLS-DA loading S-plots that contributed to the observed class discrimination in both WT and mutants were annotated and putatively identified.

### 2.3. Metabolite Annotation and Functional Classification

**Figure 3:**
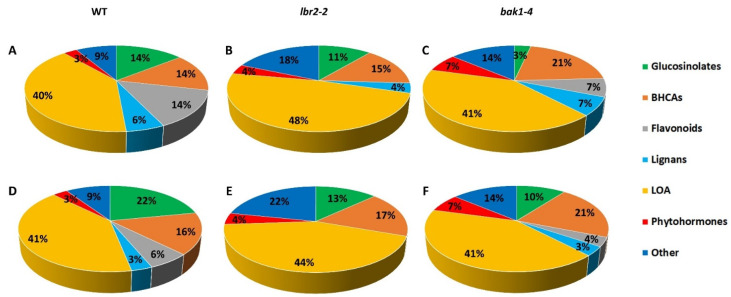
LPS-induced discriminant metabolite classes identified in Arabidopsis WT and mutant (*lbr2-2* and *bak1-4*) lines (Table 1). (**A**–**C**) represents discriminant metabolites from LPS*_Pst_*-treated WT, *lbr2-2* and *bak1-4*, respectively. (**D**–**F**) represents discriminant metabolites from LPS*_Xcc_* -treated WT, *lbr2-2* and *bak1-4*, respectively. These metabolites contribute to the defence response in Arabidopsis WT and mutants. Abbreviations: BHCAs = benzoic- and hydroxycinnamic acid derivatives; LOA = lipids, oxylipins and arabidopsides.

Forty-five statistically significant LPS-induced metabolites were annotated and putatively identified according to level 2 of the Metabolomic Standard Initiative (MSI) [26], and listed (Table 1 and Table S1) for the metabolites and their respective diagnostic fragmentation patterns, as well as KEGG IDs. The identified metabolites were grouped according to their functional categories: glucosinolates, benzoic- and HCA derivatives, flavonoids, lignans, lipids, oxylipins, arabidopsides, phytohormones, and other metabolite classes (Table 1; Figure 3). Combined, LPS*_Pst_* induced accumulation of 35, 27 and 30 significant metabolites in WT, *lbr2-2* and *bak1-4*, respectively, whereas LPS*_Xcc_* triggered the accumulation of 32, 23 and 29 significant metabolites in WT, *lbr2-2* and *bak1-4*, respectively. These metabolites were either shared amongst the plants (common) or differentially detected in the WT, *lbr2-2* or *bak1-4* (Table 1). Similarly, all the metabolite classes identified from LPS*_Pst_*- or LPS*_Xcc_*-treated Arabidopsis WT, *lbr2-2* or *bak1-4* were presented as percentages using pie charts (Figure 3). For instance, with both LPS*_Pst_* and LPS*_Xcc_*, the glucosinolate metabolite 8-(methylsulphinyl)octyl cyanide (8-MeSO-octyl-CN) [#1] was present as a discriminatory ion in WT and *bak1-4*, but not in *lbr2-2* plants. On the other hand, 8-(methylsulphinyl)octyl isothiocyanate (hirsutin) [#2] was absent in *bak1-4*, but was identified in WT and *lbr2-2* under both treatments. Of the plants under investigation, it was only in *bak1-4* treated with LPS*_Pst_* that 7-methylsulphinylheptyl isothiocyanate [#3] and 8-(methylsulphinyl)octylamine (8-MeSO-octyl-NH2) [#4] were not identified. Notably, the remaining significantly identified glucosinolates such as 4-methylthiobutyl glucosinolate (glucoerucin) [#5] (only in LPS*_Xcc_*), 3-indolylmethyl glucosinolate (glucobrassicin) [#6] (only in LPS*_Xcc_*) and 8-methylsulfinyloctyl glucosinolate (glucohirsutin) [#7] (in both LPS chemotypes treatment) were only detected in WT, but not in the two mutant lines. Overall, in WT, *lbr2-2* and *bak1-4* treated by LPS*_Pst_*, the percentage of glucosinolates identified were 14, 11 and 3%, respectively (Figure 3A–C). In comparison, 22, 13 and 10% of glucosinolates were detected when the different plants were treated with LPS*_Xcc_* (Figure 3D–F). Moreover, the percentage of glucosinolates were dominant in WT treated with both LPS chemotypes followed by *lbr2-2* and *bak1-4*, respectively.

Hydroxycinnamic acid-derivatives such as 6,7-dimethoxycoumarin (scoparone) [#8], sinapic acid [#9] and sinapoyl malate [#10] were identified in the Arabidopsis WT, *lbr2-2* and *bak1-4* for both LPS chemotypes treatments, while 1-O-sinapoyl-beta-D-glucose [#13] was detected in the WT and *bak1-4* (both chemotypes), but not in *lbr2-2*. Furthermore, benzoic acid derivatives such as 2,5-dihydroxybenzoic acid pentoside isomer I [#11] accumulated in WT, *lbr2-2* and *bak1-4* (for both chemotypes), whereas 2,5-dihydroxybenzoic acid pentoside isomer II [#12] was detected in *bak1-4* only for both LPS chemotypes treatments. The percentage of benzoic- and HCA derivatives identified in WT, *lbr2-2* and *bak1-4* after LPS*_Pst_* treatment were 14, 15 and 21%, respectively, while 16, 17 and 21% of the same group of metabolites were detected after LPS*_Xcc_* treatment (Figure 3). Overall, the percentage of LPS-induced benzoic- and HCA derivatives was lowest in the WT, followed by *lbr2-2* and *bak1-4*, respectively.

Interestingly, flavonoids were not identified as discriminant markers in the *lbr2-2* mutant plants after treatment with both LPS*_Pst_* and LPS*_Xcc_* (Table 1; Figure 3). LPS*_Pst_*-treated WT and *bak1-4* had 14 and 7% flavonoids, whereas under LPS*_Xcc_* treatment the percentages were 6 and 4%, respectively (Figure 3). Most of the identified flavonoids are afzelin (kaempferol-3-rhamnoside) [#14], robinin (kaempferol-3-O-robinoside-7-O-rhamnoside [#15], kaempferitrin (kaempferol 3,7-dirhamnoside) [#16], kaempferol 3-O-rhamnoside-7-O-glucoside [#17] and 2’,4’,4-trihydroxy-3’-prenylchalcone [#18]. In all, WT accumulated the highest percentage of flavonoids, followed by *bak1-4*, but none in *lbr2-2* post-LPS treatments. Afzelin and robinin were identified at 24 h LPS*_Pst_*-treated WT. Kaempferitrin accumulated in WT treated with both LPS chemotypes, and also in the LPS*_Pst_*-treated *bak1-4* at 24 h. Additionally, kaempferol 3-O-rhamnoside-7-O-glucoside was detected only at 24 h in WT treated with both chemotypes, while 2’,4’,4-trihydroxy-3’-prenylchalcone accumulated in WT and *bak1-4* treated with LPS*_Pst_* at 18 h. In summary, flavonoids identified in this study were mostly induced by the LPS*_Pst_*, and mainly in the WT.

None of the identified lignans was observed in LPS*_Xcc_*-treated *lbr2-2*, with 3% each in WT and *bak1-4* (Table 1; Figure 3A–C), while the percentage of lignans identified in WT, *lbr2-2* and *bak1-4* treated with LPS*_Pst_* were 6, 4 and 7%, respectively (Figure 3D–F). G(8-O-4)G hexoside [#19] was only detected in WT treated with LPS*_Xcc_* at 24 h, as well as in LPS*_Pst_*-treated *bak1-4* at 18 h. Lariciresinol hexoside [#20] was detected in LPS*_Pst_*-treated WT and also in *bak1-4* treated with both LPS chemotypes (Table 1). Another lignan, G(8–5)FA malate [#21], was not identified in *lbr2-2* and *bak1-4*, but was accumulated in LPS*_Pst_*-treated WT (Table 1).

The “lipids, oxylipins and arabidopsides” (LOA) class of metabolites were abundantly identified in WT, *lbr2-2* and *bak1-4*, with few in absence (Table 1). For example, the following metabolites were present in all plants: methyl 8-hydroxy-11E,17-octadecadien-9-ynoate [#22], 9,12,13-trihydroxy-10,15-octadecadienoic acid [#23], 9,12,13-trihydroxyoctadec-10-enoic acid (9, 12, 13-TriHOME) [#24], 13S-hydroperoxy-9Z, 11E, 15Z octadecatrienoic acid (13(S)-HPOTrE) [#25] (for both chemotypes, but not in LPS*_Pst_*-treated *bak1-4*), 7S,8S-dihydroxy-9Z,12Z-octadecadienoic acid (7S,8S-DiHODE) [#26], 12-oxo-phytodienoic acid (12-OPDA) [#32], and dinor-12-oxo phytodienoic acid (dinor-OPDA) [#33]. On the other hand, methyl 9,12-dihydroxy-13-oxo-10-octadecenoate [#27] was absent in both mutants under both LPS chemotype treatments but was present in WT at 18 h for both chemotypes, while 3’-O-linolenoylglyceryl 6-O-galactopyranosyl-galactopyranoside isomer I [#28] was only detected in *bak1-4* after LPS*_Pst_* treatment. Arabidopside A [#30] was not identified in either of the mutants, but only in WT for both chemotypes, whereas Arabidopside D [#31] was only identified in WT and *lbr2-2* treated by LPS*_Pst_*, but not in *bak1-4*. *Sn*2-O-(dinoroxophytodienoyl)-digalactosyl isomer II [#36] was detected in both mutants (both chemotypes) but not in WT, while *Sn*2-O-(dinoroxophytodienoyl)-digalactosyl isomer I [#35] was absent in WT treated with LPS*_Pst_*, as well as LPS*_Xcc_*-treated *lbr2-2*, but present in *bak1-4* for both chemotype treatments. Metabolites from Arabidopsis WT, *lbr2-2* and *bak1-4* treated with LPS*_Pst_* were dominated by the group “lipids, oxylipin and arabidopsides”, with the percentage of 40, 48 and 41%, respectively (Figure 3A–C). A similar trend was observed when LPS*_Xcc_* was used to treat WT, *lbr2-2* and *bak1-4*, with the said metabolites group documented as 41, 44 and 41%, respectively (Figure 3D–F). Overall, *lbr2-2* treated with LPS*_Pst_* and LPS*_Xcc_* resulted in the highest number of LOA metabolites compared to the WT and *bak1-4*.

The phytohormone abscisic acid [#39] was present as a discriminatory marker in both LPS*_Pst_*- and LPS*_Xcc_*-treated WT, *lbr2-2* and bak*1-4*, while salicylic acid 2-O-beta-D-glucoside [#40] was detected only in *bak1-4* treated with both LPS chemotypes (Table 1). Amongst “other” identified metabolites, L-threonine [#41] was identified in both LPS*_Pst_*- and LPS*_Xcc_*-treated mutants, but not in the WT, while sulforaphane-glutathione [#45] was identified only in *lbr2-2* treated with both LPS chemotypes. The remaining other metabolites [#42, 43, 44] were identified in all the plants and both LPS treatments.

**Figure 4:**
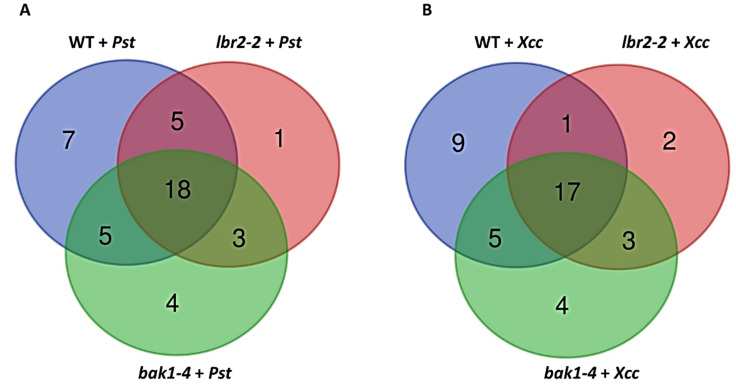
Venn diagrams of statistically significant annotated metabolites in LPS-induced Arabidopsis. (**A**) represents metabolites accumulated in the LPS*_Pst_* -treated Arabidopsis WT, *lbr2-2* and *bak1-4*. (**B**) represents metabolites accumulated in the LPS*_Xcc_*-treated Arabidopsis WT, *lbr2-2* and *bak1-4*. Diagrams show discriminatory metabolites (Table 1) that are shared and/or distinct after different LPS chemotype treatments.

Furthermore, Venn diagrams were constructed to visualise the number of LPS-induced metabolites that overlapped or were unique for Arabidopsis WT, *lbr2-2* and *bak1-4* (Figure 4). LPS*_Pst_* induced seven unique metabolites in the WT compared to one and four observed in *lbr2-2* and *bak1-4*, respectively (Figure 4A). For the LPS*_Xcc_*-treated plants, nine, two, and four metabolites were unique in the WT, *lbr2-2* and *bak1-4*, respectively (Figure 4B). Eighteen and seventeen metabolites were overlapped in LPS*_Pst_*- and LPS*_Xcc_*-treated WT, *lbr2-2* and *bak1-4*, respectively. Arabidopsis WT and *bak1-4* shared five metabolites in both LPS*_Pst_* and LPS*_Xcc_* treatments. Similarly, three metabolites were overlapped between *lb2-2* and *bak1-4* in both LPS*_Pst_* and LPS*_Xcc_* treatments. Five metabolites were overlapped between WT and *lb2-2* after LPS*_Pst_* treatment, as compared to one metabolite shared between them after LPS*_Xcc_* treatment.

From the classes of identified annotated metabolites, it is evident that the metabolomes of the Arabidopsis WT and mutants (*lbr2-2* and *bak1-4*) were reprogrammed to a defence state upon LPS treatments. Furthermore, the presence and absence of certain metabolites as observed in the WT and mutants suggest that they responded differently to the two LPS chemotype treatments as a consequence of the functional LBR2 and BAK1 proteins in WT, but not in the respective mutants.

### 2.4. Metabolite Heat Map Visualization and Analysis

**Figure 5:**
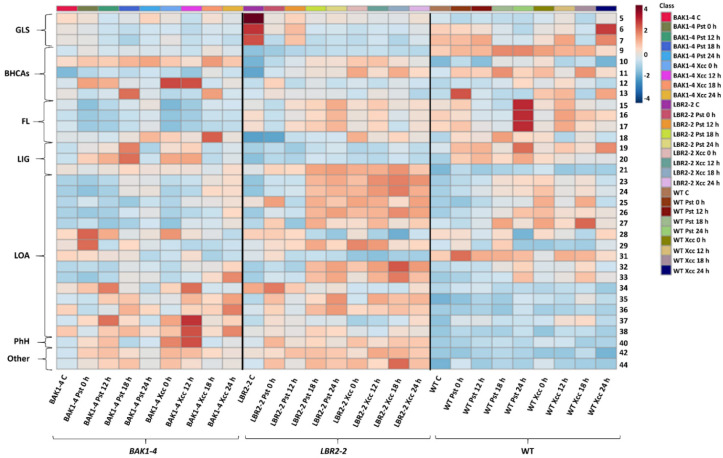
Heatmap presentation of significant annotated metabolites in ESI (−) mode. The LPS*_Pst_*– and LPS*_Xcc_*-induced annotated metabolites data of WT, *lbr2-2* and *bak1-4* were submitted to MetaboAnalyst and the relative intensities showing the extent of metabolites accumulation. The rows represent the group of the identified metabolites (numbered as in Table 1), while the columns represent Arabidopsis plants with their respective LPS chemotype time-related treatments. The colour gradient of dark blue indicates lowest intensity, while deep red indicates highest intensity. Metabolites are numbered as in Table 1. Abbreviations: GLS = glucosinolates; BHCAs = benzoic- and hydroxycinnamic acid derivatives; FL = flavonoids; LOA = lipids, oxylipins and arabidopsides; PhH = phytohormones.

Significant metabolites from LPS-induced Arabidopsis WT, *lbr2-2* and *bak1-4* were submitted to the colour-coded heat map module in the MetaboAnalyst web-based tool, to visualise and analyse the time course of relative intensities across control, 0, 12, 18, and 24 h. Notably, the heat maps display the relative intensities of the annotated metabolites across all time points. In the constructed heat maps (Figure S6 for ESI (+) data and Figure 5 for ESI (−) data), the rows represent the group of the identified metabolites, while the columns represent Arabidopsis plants with their respective LPS chemotype time-related treatments. The colour gradient of dark blue indicates the lowest concentration (relative peak intensity), while deep red indicates the highest concentration. In this study, the annotated metabolites showed a variation of metabolite accumulation as displayed by the average relative intensities (blue and red colour gradient). For the ESI (+) data, high intensities were observed within the *lbr2-2* (toward the middle and lower sections for both LPS chemotypes), followed by the *bak1-4* (towards the left for LPS*_Pst_* and towards the right for LPS*_Xcc_*), and finally to the WT (right side of the heat map). The metabolites on the top half of WT showed higher intensity (mainly glucosinolates) than the bottom, while in the *lbr2-2,* the bottom half (mainly the LOA and other metabolites) were more intense than the top half (and clearly so compared to the WT), whereas in the *bak1-4,* intensities were relatively equal in the top and bottom half (Figure S6). Metabolites identified from WT treated with LPS*_Pst_* appeared to have late increases in intensities compared to the plants treated with LPS*_Xcc_* that displayed early intensity increases (Figure S6). A similar pattern was observed in the *lbr2-2* and *bak1-4* plants, but with higher increased intensities. For the ESI (−) data, similar trends were observed where metabolites on the top half (mainly glucosinolates, benzoic acid and HCA derivatives and flavonoids) of WT had higher intensities than the bottom half. In *lbr2-2*, the bottom half (mainly the LOA, phytohormones and other metabolites) was higher in intensity than the top half, and notably so compared to the WT. Finally, in the *bak1-4*, relatively similar intensities were observed on the top and bottom half of the heat map (Figure 5).

Overall, the observed variation in intensity from the heat map analysis further revealed the differential changes in the metabolome of the Arabidopsis WT and mutants (*lbr2-2* and *bak1-4*) after different LPS chemotype treatments. More so, increased intensities observed in the LPS-treated mutants, especially the LOA metabolites group of *lbr2-2,* indicate an inducible response to the LPS chemotypes. The nature of these metabolomic responses seems different when compared to that of the WT as reflected by the greater peak intensities of defense-associated metabolites. This might suggest the presence of other LPS-interacting proteins/co-receptors other than LBR2/BAK1, or possibly the participation/complementation by close homologues such as LBP/BPI related-1 (LBR1)/BAK1-like 1 (BKK1).

### 2.5. Pathway Mapping and Quantification

**Figure 6:**
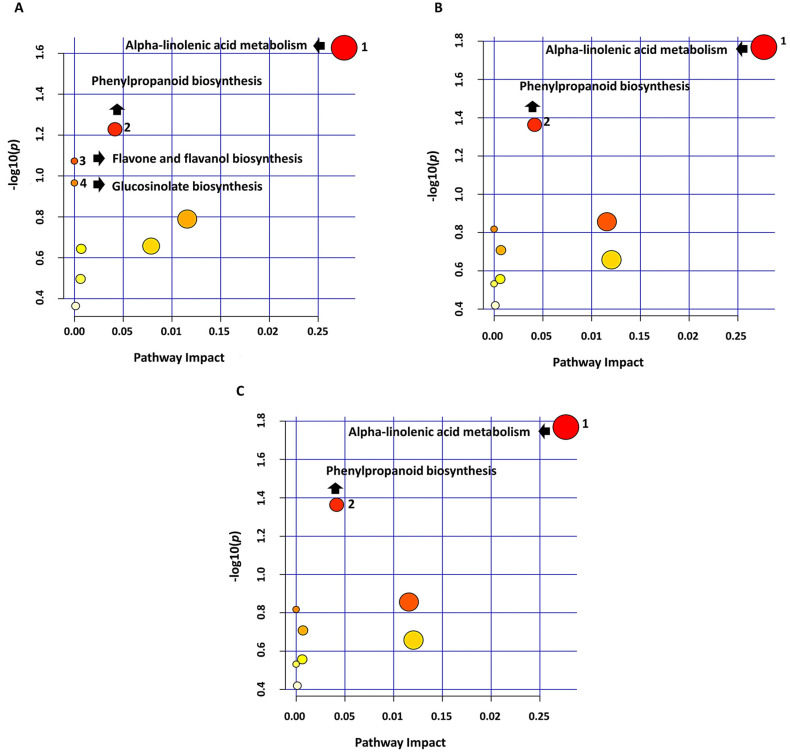
Metabolome view of metabolic pathways (MetPA) mapped from identified metabolites. Metabolite KEGG IDs from both LPS chemotype-treated WT, *lbr2-2* or *bak1-4* were mapped to pathways as displayed. (**A**–**C**) represents the metabolome view for the WT, *lbr2-2* and *bak1-4*, respectively. The graphs were arranged such that the *p*-values from the pathway analysis are on the *y*-axis and the impact values from the pathway topology analysis on the *x*-axis. The colour gradient of the circles (pathways) from light yellow to dark red indicates lower to higher significance, respectively. Pathways are numbered #**1**: Alpha-linolenic acid metabolism, #**2**: Phenylpropanoid biosynthesis, #**3**: Flavone and flavanol biosynthesis and #**4**: Glucosinolate biosynthesis. Notably, pathways **#3** and **#4** were not mapped in the *lbr2-2* and *bak1-4* mutants.

To elucidate various pathways associated with the identified LPS-induced metabolites in WT, *lbr2-2* and *bak1-4* mutants, the Metabolomic Pathway Analysis (MetPA) tool, a component of the MetaboAnalyst suite, was used. Notably, MetPA mapped only metabolites with KEGG IDs and graphically presented the data as the metabolome view and a table that contains all the altered/perturbed pathways (Figure 6A–C; Tables S2–S4).

In WT, *lbr2-2* and *bak1-4* treated with LPS*_Pst_* and LPS*_Xcc_*, alpha-linolenic acid metabolism (#1) was the most significantly altered pathway followed by the phenylpropanoid biosynthesis (#2). The flavone and flavanol biosynthesis pathway (#3) and glucosinolate biosynthesis (#4) were affected/induced in the LPS-treated WT, not in the *lbr2-2* and *bak1-4* mutants. The rest of the enriched pathways perturbed by LPS in both Arabidopsis WT and mutants are listed in Tables S2–S4. More so, the bar graphs (Figures S7–S10) display the relative intensities of the mapped metabolites (and some unmapped metabolites) of affected pathways across all time points. These results, as elaborated below, suggest that Arabidopsis uses different pathways to reprogramme its metabolome into a defensive state when challenged by LPS MAMPs. The relative quantification of the average intensities of metabolites from the selected four pathways: alpha-linolenic acid metabolism (#1), phenylpropanoid biosynthesis (#2), flavone and flavanol biosynthesis pathway (#3), glucosinolate biosynthesis (#4), are shown (Figures S7–S10).

Figure S7A,B show the alpha-linolenic acid metabolism mapped metabolites, 13S(S)-HPOTrE and 12-OPDA with the relative intensities. The 13S(S)-HPOTrE had increased intensity in the WT treated with LPS*_Pst_* and LPS*_Xcc_*. There were increased intensities of 13S(S)-HPOTrE in *lbr2-2* with reduced intensities at 12 h LPS*_Pst_* treatment (Figure S7A). The 13S(S)-HPOTrE in *bak1-4* decreased in intensity after 0 h, but picked up at 24 h when treated by LPS*_Pst_*. On the other hand, when treated with LPS*_Xcc_*, there was a steady increase in intensity from 12 to 24 h. In all, there was an overall greater intensity of 13S(S)-HPOTrE in WT than in the mutants, when comparing the control vs. treatments. For 12-OPDA identified in WT, there was an increase in intensities across treatments with LPS*_Pst_* and LPS*_Xcc_*, with the highest intensities observed at 24 and 18 h, respectively (Figure S7B). In *lbr2-2,* there was an increase in 12-OPDA intensities at 18 to 24 h after treatment by LPS*_Pst_*. On the other hand, the LPS*_Xcc_* treatment showed a steady increase in intensities of 12-OPDA at early and late time points. The 12-OPDA in *bak1-4* plants showed mainly late accumulation in both LPS*_Pst_* and LPS*_Xcc_* treatments. Overall, 12-OPDA identified in *lbr2-2* appeared to have the highest intensities followed by WT and *bak1-4*, across all treatments. The second altered pathway was phenylpropanoid biosynthesis with the analysed intensities of the two mapped metabolites (sinapic acid and sinapoyl malate) (Figure S8A,B). The intensity of another identified phenylpropanoid metabolite sinapoyl 1-O-sinapoyl-beta-D-glucose that was not mapped by MetPA was included in the analysis (Figure S8C). Figure S8A shows that the relative intensities of sinapic acid identified from WT after LPS*_Pst_* treatment increased exponentially, but decreased when treated with LPS*_Xcc_*. A similar trend was observed in *lbr2-2* plants but with reduced relative intensities. The relative intensities of metabolites from *bak1-4* after LPS treatment appeared to have similar intensities across the time points (Figure S8A). For sinapoyl malate (Figure S8B), there was an increase in intensity in both early and late responses after LPS*_Pst_* treatment, but a reduced late response at 24 h LPS*_Xcc_* treatment. In *lbr2-2*, sinapoyl malate had both early and late increased intensities with a slight reduction when treated with LPS*_Xcc_* at 12 h. Sinapoyl malate from *bak1-4* also had both early and late increased intensities with a slight reduction when treated with LPS*_Xcc_* at 12 h.

For 1-O-sinapoyl-beta-D-glucose (Figure S8C), the overall intensities of the WT and mutant plants decreased significantly (over 10-fold) when compared to those of the sinapic acid and sinapoyl malate. The WT treated with LPS*_Pst_* had an early increase but reduced significantly after 12 h to 24 h. This was not the case for the LPS*_Xcc_*-treated WT that only had reduced intensity at 18 h. The 1-O-sinapoyl-beta-D-glucose accumulated in *lbr2-2* had increased intensities across all time points after LPS*_Pst_* treatment, but in 12 h and 18 h after in LPS*_Xcc_* treatments. For the *bak1-4*, there was an increase of intensity at 18 h LPS*_Pst_* and LPS*_Xcc_* treatments.

For the flavone and flavanol biosynthesis pathway, kaempferol-3-O-rhamnoside-7-O-glucoside was mapped (Figure S9A). The average intensity of two other flavonoids (afzelin and kaempferitrin) that were not mapped by MetPA were also analysed (Figure S9B,C). There was increased accumulation of kaempferol-3-O-rhamnoside-7-O-glucoside accumulation in WT at 24 h and 12 h LPS*_Pst_* and LPS*_Xcc_* treatments, respectively (Figure S9A). There was no clear increase in the accumulation of kaempferol-3-O-rhamnoside-7-O-glucoside in *lbr2-2* and *bak1-4*, albeit a slight increase at the 24 h LPS*_Pst_*-treated *lbr2-2*. For afzelin (Figure S9B), there was a clear increase of intensity at 24 h and 12 h after LPS*_Pst_* and LPS*_Xcc_* treatments, respectively, in WT; whereas there was no clear increase observed in *lbr2-2* and *bak1-4* after both LPS chemotype treatments, albeit a slight increase observed at 12 h and 24 h LPS*_Xcc_* and LPS*_Pst_* treatments in *lbr2-2* and *bak1-4*, respectively. For the kaempferitrin, an increase of intensity was observed in 24 h LPS*_Pst_*-treated WT, with no clear increase in *lbr2-2* and *bak1-4* after both LPS*_Pst_* and LPS*_Xcc_* treatments. Notably, the flavone and flavanol biosynthesis pathway was seemingly not affected by the LPS*_Pst_* and LPS*_Xcc_* chemotypes.

Furthermore, the glucosinolate biosynthesis pathway mapped glucoerucin and glucobrassicin metabolites and their relative intensities are shown in Figure S10A,B, respectively. In addition, the intensities of MetPA unmapped glucosinolates such as glucohirsutin and glucosinolate degradation product 8-(methylsulphinyl)octylamine were analysed in Figure S10C,D, respectively. Glucoerucin identified in LPS*_Xcc_*-treated WT plants showed a slight decrease in intensities across time points when treated with LPS*_Pst_*, but with a slight increase at 12 h and 24 h with LPS*_Xcc_*. For *lbr2-2*, there was over a 2-fold decrease of intensities across time points in both LPS*_Pst_* and LPS*_Xcc_*-treated plants. Glucoerucin from *bak1-4* showed decreased and similar intensities in both LPS chemotypes treatments. For glucobrassicin (Figure S10B), there were decreased intensities in the WT treated with LPS*_Pst_* and LPS*_Xcc_* across time points, except at 24 h LPS*_Xcc_* treatment where there was, relatively, over a 1.5-fold increase in intensity. In *lbr2-2*, glucobrassicin showed over a 1.6- and 2.5-fold decrease in intensity when treated with LPS*_Pst_* and LPS*_Xcc_*, respectively. Glucobrassicin in *bak1-4* treated with LPS*_Pst_* and LPS*_Xcc_* showed decreased and similar intensities when comparing the control vs. treatment. Glucohirsutin (Figure S10C), was increased at 12 h and 24 h LPS*_Pst_* and LPS*_Xcc_*-treated WT, but decreased in both *lbr2-2* and *bak1-4*. The glucosinolate degradation product 8-(methylsulfinyl)octylamine was accumulated in both WT, *lbr2-2* and *bak1-4* after both LPS chemotypes treatments, with lesser accumulation observed in LPS*_Pst_*-treated *bak1-4* (Figure S10D).

Overall, LPS chemotypes altered the glucosinolate biosynthesis pathway and led to more accumulation of glucosinolates and their products in the WT as compared to the mutants.

## 3. Discussion

LPSs found on the outer membrane of Gram-negative bacteria is one of the MAMPs associated with bacterial adhesion and induction of defence responses in animals and plants [28]. The structure of LPS is made up of lipid A, core oligosaccharides and an O-polysaccharide chain, which have been reported to induce defence responses in plants individually or collectively [4,29,30]. To date, the plant pattern recognition receptor (PRR) and/or co-receptor involved in LPS perception, as well as the molecular mechanism underlying the defence signalling, remains elusive. Sanabria et al. [3] have proposed the involvement of an LPS-responsive *N. tabacum* S-domain RLK (*Nt-Sd-RLK*) gene encoding conserved B-lectin, S- and PAN domains in LPS perception. Additionally, a recent study implicated Arabidopsis AtLBR-1 and AtLBR-2 in the LPS perception and defence responses [4].

In this study, untargeted metabolomics were used to profile the metabolite changes that might occur after treatment of Arabidopsis WT and mutant (*lbr2-2* ad *bak1-4*) plants with different LPS chemotypes from *Pseudomonas syringae* pv. *tomato* DC3000 (*Pst*) and *Xanthomonas campestris* pv. *campestris* 8004 (*Xcc*). Advancement in liquid chromatography-mass spectrometry (LC-MS) has improved the use of metabolomics in the study of MAMP-treated plants [11,12,31]. Here, chromatographic and spectrometric changes were observed in extracts from both Arabidopsis WT and the mutants after treatments with LPS*_Pst_* and LPS*_Xcc_.* Such perturbations can be linked to the metabolome changes caused by LPS chemotypes treatments. The 12–24 h period selected in this study has been shown to be sufficient to capture the metabolome changes that may occur and possibly return the plant system to homeostasis [12,31]. The chemometric modelling tools such as PCA and OPLS-DA play vital roles in the observed sample grouping and accompanying metabolite identification that reflects on the evidence of differential metabolic reprogramming that is related to the response of Arabidopsis plants to the LPS chemotypes. There were broad classes of annotated metabolites involved as significant response markers, including glucosinolates, benzoic acids and HCAs, flavonoids, lignan, lipids, oxylipins, arabidopsides and phytohormones, that unravelled the complexity of the plant metabolomes and the implication of LPS in the Arabidopsis defence responses.

### 3.1. Glucosinolates Accumulation Following LPSs Treatment

Glucosinolates are a sulphur-rich, structurally diverse class of secondary metabolites that are richly found in Brassicaceae such as Arabidopsis, with various roles that include response against plant pathogens [32]. In this regard, the glucosinolate–myrosinase defence system is activated upon pathogen or insect attack in plants. The glucosinolate breakdown activity of myrosinase leads to the production of various degradation products that are involved in the defence activities [33]. In this study, we annotated and putatively identified a number of glucosinolates and their products in both Arabidopsis WT and mutants (*lbr2-2* and *bak1-4*) (Table 1). However, WT treated with LPS*_Pst_* and LPS*_Xcc_* accumulated more glucosinolates compared to the *lbr2-2* and *bak1-4*. Similar trends were observed when glucosinolates (e.g., glucoerucin and glucobrassicin) were mapped by MetPA. Consistently, there were higher qualitative accumulation of glucoerucin, glucobrassicin and glucohirsutin when comparing control vs. LPS treatments in the WT vs. *lbr2-2* and *bak1-4* (Figure S10). While glucosinolates were identified only in the WT, glucosinolate degradation products were identified in both WT and mutants. For instance, glucosinolate degradation product 8-(methylsulfinyl)octylamine accumulated in both LPS*_Pst_*- and LPS*_Xcc_*-treated WT, *lbr2-2* and *bak1-4*. Robin et al. [34] reported increased accumulations of both glucoerucin (e.g., of aliphatic glucosinolate) and glucobrassicin (e.g., of indole glucosinolate) in the resistant cabbage line BN4303 infected by *Leptosphaeria maculans* that causes blackleg disease in *Brassica* crops. Furthermore, aliphatic and aromatic glucosinolates enhanced resistance against necrotrophic bacteria *Erwinia caratovora* and hemi-biotrophic bacteria *P. syringae*, respectively, in Arabidopsis [35]. These studies highlight the significant role of glucosinolates in plant defence against pathogens. In our group, several glucosinolates including glucohirsutin, glucoerucin, glucobrassicin were putatively annotated from Arabidopsis treated by LPS from *B. cepacia* [11,12]. The results obtained in this study (both the annotated metabolites, heat map and MetPA analyses) suggested that different LPS chemotypes induced significant accumulation of glucosinolates in the WT compared to the mutants. Moreover, the variation in the accumulation of glucosinolates and their degradation products in the WT compared to the mutants may be caused by the knock out of the LBR2 and BAK1 proteins.

### 3.2. Hydroxycinnamic Acid Derivatives Accumulation Post-LPSs Treatment

HCAs and their derivatives are indispensable in the protection of plants against pathogens, ultraviolet B irradiation and central to the synthesis of secondary metabolites such as flavonoids, tannins and lignin [36,37]. The synthesis of HCAs and derivatives from phenylalanine through the phenylpropanoid pathway is well-documented in plants, including the Arabidopsis model system [38]. In this study, LPS*_Pst_* and LPS*_Xcc_* triggered the differential accumulation of metabolites that belong to the HCA derivatives such as 6,7-dimethoxycoumarin, sinapic acid and sinapoyl malate in WT and the mutants. For example, 1-O-sinapoyl-beta-D-glucose was detected in WT and *bak1-4*, but not in the *lbr2-2*, suggesting its differential accumulation that may be linked to the absence of LBR2 protein. 

Sinapic acid may be found in free form or esterified like other HCAs. These esters can be sugar conjugates (glycosides) such as sinapoyl glucose (identified in this study), or conjugates of other compounds, such as sinapoyl malate (identified in this study) and sinapoyl choline or sinapine (a source of sinapic acid and choline in germinating mustard seed) [39,40,41,42]. Tinte et al. [11] also identified sinapic acid, sinapoyl glucose and sinapoyl malate in Arabidopsis after treatment with LPS from *B. cepacia* Furthermore, the antibacterial activity of sinapic acid against both Gram-positive and Gram-negative bacteria have been reported in mustard seeds [43,44]. Sinapoyl glucose was one of the infection markers identified in the apoplasts of Arabidopsis leaves infected by *Verticillium longisporum* [45]. An Arabidopsis mutant deficient in sinapoyl malate or sinapic acid affected the accumulation of sinapic esters and disrupted the phenylpropanoid pathway and eventual lignin biosynthesis [40,46]. Heat map (Figure 5) and MetPA analysis (Figure 6) showed that the phenylpropanoid biosynthesis pathway was altered with variations in the accumulation of sinapic acid, sinapoyl malate and sinapoyl 1-O-sinapoyl-beta-D-glucose when the control vs. LPS treatments were compared (Figure S8). These results showed increased accumulation of HCAs and derivatives in WT compared to the mutants (*lbr2-2* and *bak1-4*), further highlighting a possible influence of different LPS chemotypes and lack of functional LBR2-2 and BAK1-4 proteins in the perturbation observed in the Arabidopsis metabolome.

### 3.3. Flavonoids Accumulation Post-LPSs Treatment

Flavonoids are secondary metabolites produced in plants through the phenylpropanoid pathway with a variety of functions that include defence against environmental stresses, pigmentation, auxin transport, signalling, feeding deterrents and protection against UV light [47,48,49]. As shown in Table 1, more flavonoids were accumulated in WT followed by *bak1-4*, with none identified in the *lbr2-2*. Flavonoids accumulated mostly at 24 h post-LPS treatment in the WT suggesting a late flavonoid response in Arabidopsis. The absence of significant flavonoids in the *lbr2-2* suggests a possible role of LBR in the LPS perception Arabidopsis. MetPA analysis (Figure 6) showed the mapping of kaempferol 3-O-rhamnoside-7-O-glucoside in the altered flavone and flavonol biosynthesis pathway. This LPS-induced kaempferol 3-O-rhamnoside-7-O-glucoside and unmapped flavonoids such as afzelin and kaempferitrin displayed distinct variations in quantitative intensities, suggesting differential metabolic accumulation in the WT and mutants. Kaempferol is involved in the defence response of Arabidopsis against the Cucumber mosaic virus containing satellite RNA (CMVsat) infection [50]. Kaempferitrin is reported as the key flavonoid that inhibits the polar auxin transport (PAT) and thereby regulates growth and development in Arabidopsis shoots [51]. A study of prenylated flavonoids showed high free radical scavenging activities that contribute to their use as potent anti-oxidants [52].

### 3.4. Lignan Accumulation Following LPSs Treatment

Lignans are synthesised from HCAs through the early phenylpropanoid pathway with precursors such as coniferyl alcohol, sinapyl alcohol and 4-hydroxycinnamyl alcohol [53]. Lignan biosynthesis is related to but different from that of phenylpropanoids such as lignins, norlignans and neolignans [54,55,56]. There is evidence of bacteria and fungi triggering accumulation of lignin and lignan, leading to the protection of plants against pathogen invasion [46,57]. Cinnamyl alcohol dehydrogenase (CAD) genes that code for the enzyme that catalyses the final step in the biosynthesis of monolignol have been shown to accelerate lignin and lignan biosynthesis and deposition are induced following pathogen infection [58]. Lignans such as G(8-O-4)G hexoside, lariciresinol hexoside and G(8–5)FA malate were identified in response to LPS treatment in this study (Table 1).

The fungal pathogen *V*. *longisporum* induced accumulation of phenylpropanoid metabolites including lignans such as the syringaresinol glucoside (a sinapyl alcohol-derived lignan), secoisolaricoresinol, pinoresinol glucoside and lariciresinol glucoside (coniferyl alcohol-derived lignans) in Arabidopsis [46]. G(8-O-4)G hexoside, G(8–5)FA malate and lariciresinol hexoside identified in this study were reported to accumulate in Arabidopsis treated with silver nanoparticles [59]. In this study, the accumulation of lignans upon LPS treatment was higher in the WT followed by *bak1-4* and decreased in *lbr2-2* (from heat map analysis). This supports the above suggestions that different LPS chemotypes are able to trigger this metabolic pathway, as well as that the BAK1 and LBR2 proteins play a possible regulatory role in the accumulation of lignan. These findings further correlate with existing reports that link lignans in plant defence against biotic and abiotic stresses.

### 3.5. Lipids, Oxylipins and Arabidopsides Accumulation Post-LPSs Treatment

Lipids are involved in the establishment of membrane interfaces as well as in the regulation of intracellular signalling during plant–microbe communication [60,61]. Oxidation products of polyunsaturated fatty acids (PUFAs) known as oxylipins function as signal molecules in the regulation of plant growth, development, and biotic and abiotic responses [60,62]. Fatty acid derivatives such as 9,12,13-trihydroxy-10,15-octadecadienoic acid and 9,12,13-TriHOME detected in this study have been identified from the Arabidopsis root exudate profile [63] and the apoplast of Arabidopsis infected by *V*. *longisporum* [45]. Tinte et al. [11] reported accumulation of 13(S)-HPOTrE, 12-OPDA and Arabidopside A in Arabidopsis leaves treated by LPS from *P. syringae*. Arabidopsides are named based on the position at which OPDA is found esterified to the mono- or digalactosyl diacylglycerol (MGDG or DGDG), instead of the fatty acyl moiety. For instance, Arabidopside A (identified here) is a MGDG derivative containing OPDA esterified at position *sn*-1, while Arabidopside C is a DGDG containing dn-OPDA esterified at position *sn*-2 [64,65,66]. Oxylipin, 12-OPDA is a precursor for phytohormone jasmonic acid (JA) and related jasmonates such as methyl jasmonate (MeJA) and jasmonyl-L-isoleucine (JA-IIe), which are involved in plant defence responses and growth [64]. Arabidopside D (detected in this study) has previously been identified in the aerial parts of Arabidopsis with a characteristic inhibitory effect on cress root growth [67]. Additionally, *V*. *longisporum* induced accumulation of 12-OPDA and dinor-OPDA as signatory infection markers in the apoplast of Arabidopsis [45].

Our MetPA analysis implicated 13(S)-HPOTrE and 12-OPDA as mapped metabolites in the enriched alpha-linolenic acid metabolic pathway (Figure 6 and Figure S7). Both metabolites are precursors of JA with evidence in the regulation of plant defence signalling against pathogens [66]. There were distinct relative intensity changes of both 13(S)-HPOTrE and 12-OPDA metabolites when comparing control vs. LPS*_Pst-_*-and LPS*_Xcc_*-treated WT, *lbr2-2* and *bak1-4*. Overall heat map analysis showed LPS-induced more LOA metabolite group in *lbr2-2*, followed by *bak1-4* and WT. This alteration of metabolite intensities, especially LOA in mutants (mainly in *lbr2-2*), suggests that the knock-out of LBR2 and BAK1 appeared to enhance the accumulation of this group of metabolites upon LPS treatment. Overall, the result indicates that the alpha-linolenic acid metabolism is differentially altered in the Arabidopsis WT and mutants treated by different LPS chemotypes, with the WT exhibiting stricter metabolic control over LPS inducible responses/pathways.

### 3.6. Phytohormones Accumulation Following LPSs Treatment

Salicylic acid (SA) accumulates in both local and systemic defence responses against pathogens where it mediates disease resistance in both the infected and distal leaves [68,69]. SA can be synthesised through the isochorismic acid (IC) or phenylalanine ammonia lyase (PAL) pathways, using shikimic acid as a precursor in both routes [70]. Free SA level can be regulated through conversion into alternative forms by chemical modification such as methylation (e.g., methyl SA, MeSA), glycosylation (e.g., SA glucoside, SAG), and hydroxylation to form dihydroxybenzoic acids. SAG is stored in the vacuole and can be reconverted to SA when needed by the plant. SAG was identified as a disease infection marker in Arabidopsis treated with *V*. *longisporum* [45]. Furthermore, *B. cepacia* LPS induced accumulation of conjugated SA and systemic acquired resistance (SAR) against *Pst* in Arabidopsis [71].

In this study, SAG accumulated in both LPS chemotype-treated *bak1-4*, but not in the WT or *lbr2-2*. Two isomers of the benzoic acid precursors of SA such as 2,5-dihydroxybenzoic acid pentoside isomer I and II were identified in this study. While 2,5-dihydroxybenzoic acid pentoside isomer I accumulated in both LPS chemotypes-treated WT, *lbr2-2* and *bak1-4*, it was detected earlier in *bak1-4* only. The accumulation of both SAG and 2,5-dihydroxybenzoic acid pentoside isomer II in LPS-treated *bak1-4* suggests that inactive SA was more abundant in *bak1-4* mutant than WT and *lbr2-2*. While SA is involved in the defence response against biotrophic pathogens, JA regulates infection of necrotrophic pathogens or herbivorous insects, respectively, with some degree of antagonism reported between the two phytohormones [72,73].

Another phytohormone, abscisic acid (ABA), was detected in Arabidopsis treated by LPS in this study and in the study by Tinte et al. [11]. ABA accumulated in both LPS chemotypes-treated WT, *lbr2-2* and *bak1-4* with increased intensity observed in the mutants (*lbr2-2* and *bak1-4*) compared to the WT. ABA is a sesquiterpene signalling molecule that regulates pathways involved in plant responses to abiotic stresses, plant growth and development [72]. An antagonistic interplay between ABA and the JA-ethylene signalling pathway has been shown to regulate expression of plant defence genes and resistance to diseases [74].

## 4. Materials and Methods

### 4.1. Plant Growth Conditions and Genotyping

Wild type (WT) *Arabidopsis thaliana,* ecotype Columbia (Col-0) and T-DNA insertion lines (*lbr2-2* (At3g20270, SALK_132326) were obtained from Arabidopsis Biological Resource Center (ABRC) (The Ohio State University, Columbus, OH, USA), while *bak1-4* (At4g33430, SALK_116202) was a gift from Professor Thorsten Nürnberger (University of Tübingen, Baden-Württemberg, Germany). The seeds were sown in plant growth trays containing Germination Mix soil (Culterra, Muldersdrift, South Africa). All plants were grown at 23 °C, 50% humidity and under 70 µmol/m^−2^/sec fluorescent illumination in a 12 h light/12 h dark cycle. Distilled water and fertiliser (Nitrosol 1:250 (*v*/*v*)) were applied to plants once every week. Eight-week-old plants were used for experiments.

For genotyping, a two-step PCR genotyping assay, as reported in [75], with minor modifications was used to select mutant plants that were homozygous for the transferred (T)-DNA insert, and also according to the guideline tools of the Salk Institute Genome Analysis Laboratory (SIGnAL) (http://signal.salk.edu/tdnaprimers.2.html) accessed on 25 June 2018 (data not shown).

### 4.2. LPS Isolation and Characterisation

LPSs from *Pst* and *Xcc* were isolated using the hot phenol-water procedure [76], and characterised for suitable purity, as described in Tinte et al. [11] (data not shown).

### 4.3. Plant Treatment and Harvesting

LPS (dissolved to a final concentration of 100 µg/mL in sterile 2.5 mM magnesium chloride (MgCl_2_)) from *Pst* and *Xcc* was used to treat Arabidopsis WT and mutant (*lbr2-2* and *bak1-4*) plants by gentle pressure-infiltration with a blunt syringe through the abaxial side of the leaf. Three different plants were used for one biological replicate out of a total of three, and the leaves were harvested after 0, 12, 18, and 24 h post-treatments, frozen in liquid nitrogen to quench metabolic activity, and stored at –80 °C until use. Negative control plants were treated with sterile 2.5 mM MgCl_2_ to account for responses that may be generated by the MgCl_2_ solution (though it has been shown not to trigger any secondary effects [11,12]) used to dissolve the LPS, as well as possible wounding upon LPS treatment.

### 4.4. Metabolite Extraction and Sample Preparation

A methanol (MeOH)-based extraction method that extracts semi-polar and some non-polar metabolites was used to extract metabolites from the leaf tissue. Hot MeOH (Romil SpS, Cambridge, UK) (65 °C) was used to quench myrosinase activity since this enzyme degrades glucosinolate secondary metabolites that play vital roles in plant defence against environmental stresses including pathogens [12]. Hot 80% MeOH (1:10 *w*/*v*) was added to 1 g frozen leaf material in 50 mL Falcon tubes and homogenised with an Ultraturrax homogeniser (IKA, Staufen, Germany) for 2 min. For extra lysis of cells, the homogenate fraction was sonicated with a sonication probe (Sonopuls, Berlin, Germany) set at 100% power, for 30 s. The mixture was centrifuged at 5 × 300 *g* for 20 min at 4 °C and the supernatants were carefully transferred into new Falcon tubes. The supernatants were evaporated to approximately 1 mL at 55 °C using a Büchi Rotavapor R-200, transferred to 2 mL Eppendorf tubes, and then further evaporated to complete dryness using a dry bath at 55 °C in a fume hood. Dried residue was re-suspended in 500 µL of 50% ultra-high performance liquid-chromatography (UHPLC)-grade MeOH: milliQ water (1:1 *v*/*v*), filtered through 0.22 µm nylon syringe filters into HPLC glass vials fitted with 500 µL inserts and stored at 4 °C until ready for analysis. The quality control (QC) samples were prepared by pooling and mixing aliquots of equal volumes from all samples and included in the run order to monitor the stability of the samples and the instrument. Blank samples (containing 50% MeOH used to re-suspend the samples) were included in the run to gauge sample variance and monitor potential sample carryover.

### 4.5. Liquid Chromatography-Mass Spectrometry Analysis

Several LC-MS and hybrid analytical methods have shown improved sensitivity, speed and repeatability, and therefore have been used successfully in the analysis and detection of metabolome changes stemming from both biotic and abiotic stresses in plant systems [77]. In this study, samples were analysed using an Acquity UHPLC system coupled in tandem to a Synapt G1 high-definition (HD) quadrupole time-of-flight (qTOF) mass spectrometer (UHPLC-qTOF-MS) system (Waters Corporation, Manchester, UK) fitted with an Acquity HSS T3 C18 reverse-phase column (1.7 µm, 150 mm × 2.1 mm) (Waters Corporation, Milford, MA, USA). Four microliters of each sample were injected into a column that is housed in a column oven thermostatted at 60 °C. Analysis was conducted with a binary solvent system (solvent A: milliQ water and 0.1% formic acid (Sigma Aldrich, Munich, Germany); solvent B: acetonitrile (Romil SpS, Cambridge, UK) and 0.1% formic acid) at a constant flow rate of 0.4 mL/min for 30 min. The separation conditions were: 2% B over 0.00–2.00 min, increased to 90% B over 1.00–25.00 min, increased to 95% B over 25.00 min-25.10 min, held at 95% B over 25.10–27.00 min, decreased back to 2% B over 27.00–28.00 min, and held at 2% B over 28.00–30.00 min. Separated analytes were detected using a photodiode array (PDA) detector scanned from 200–500 nm with 1.2 nm resolution and a sampling rate of 20 spectra/s.

The chromatographic effluent was further analysed and detected using the MS component of the SYNAPT G1 high definition qTOF-MS, used in V-optics and operated in the electrospray ionisation positive (ESI+) and negative (ESI–) modes. The MS conditions were also set up: a capillary voltage of 2.5 kV, sampling cone voltage at 30 V, trap collision 3 V and the extraction cone at 4 V, scan time of 0.1 sec covering the 50 to 1200 Da mass-to-charge (*m*/*z*) range, source temperature of 120 °C, desolvation temperature of 450 °C. N_2_ gas was used as the nebulisation gas at a flow rate of 800 L/h. Leucine encephalin (50 pg/mL) was used as the reference mass calibrant to obtain typical mass accuracies between 1–3 mDa. The MS analysis file was set up in (MS^E^) mode to perform unfragmented as well as five fragmenting experiments simultaneously by in-source collision energy from 3–40 eV.

To ensure experimental reproducibility, three independent biological replicates and three instrumental technical replicates were analysed for each time point in a randomised fashion. This generated *n* = 9, required for the multivariate data analysis of metabolomics data [26]. As mentioned, blank and QC samples were randomly included in the run in order to monitor instrument reproducibility.

### 4.6. Multivariate Data Analysis

UHPLC-qTOF-MS raw data was visualised and processed using MarkerLynx XS^TM^ software (Waters, Manchester, UK). Parameters set to analyse ESI (+) mode data were: retention time (Rt) range of 1.1–24.1 min of the MS chromatogram, mass range of 100–1200 Da, Rt window of 0.3 min and mass tolerance of 0.05 Da. Those of ESI (−) mode data were: retention time (Rt) range of 0.9–24.0 min of the MS chromatogram, mass range of 100–1200 Da, Rt window of 0.3 min and mass tolerance of 0.05 Da. Peak detection and alignment were achieved using the MarkerLynx patented ApexPeakTrack algorithm. Normalisation was done using total ion intensities of each defined peak, while Savitzky-Golay smoothing and integration was done before computing intensities. Only data matrices with noise levels less than 50% were selected for downstream data analysis.

After data pre-processing and pre-treatment steps, the obtained data matrix of retention time (Rt)-*m*/*z* variable pairs, with *m*/*z* peak intensity for all samples, were exported to ‘Soft independent modelling of class analogy’ (SIMCA) software, version 15 (Sartorius, Umea, Sweden) for statistical modelling. The first statistical modelling used were unsupervised principal component analysis (PCA) and hierarchical clustering analysis (HiCA) dendrogram models that show trends, clusters, differences and similarities between samples. Thereafter, the statistical modelling used was a supervised model, orthogonal projection to latent structures discriminant analysis (OPLS-DA), which predefines the sample groups to observe the extent to which the variables affect the separation between groups. Selected discriminant ions for downstream metabolite identifications had a correlation [*p*(corr)] of ≥ 0.5 and covariance of (p1) ≥ 0.05. For all statistical modelling, Pareto-scaling was used to reduce the mask effect from the more abundant metabolites and, by doing so, enhance the model’s predictive ability [78]. The different model validations used to gauge model quality included evaluation of the model goodness-of-fit and predicted variation (R^2^ and Q^2^), and receiver operator classifier (ROC) plots. The OPLS-DA models were also validated using analysis of variance of the cross-validation predictive residuals (CV-ANOVA), with a *p*-value of < 0.05 indicating a good model [79]. Only statistically valid models were selected and used in data mining for metabolite annotation.

### 4.7. Metabolite Annotation and Biological Interpretation

The statistically significant metabolite annotation was performed according to level 2 of the Metabolomics Standard Initiative (MSI) [26]. The accurate mass, fragmentation patterns, elemental composition, Rt and online database searches were used to facilitate assigning the correct chemical structure to each detected feature. Using Markerlynx software, fragment ion peaks were used to obtain elemental compositions which were searched on databases for metabolites annotation. Online databases used for metabolite identification were LIPID MAPS [80], MetaCyc [81] ChemSpider [82] and Dictionary of Natural Products [83]. Mass spectral information was also compared to available published literature and metabolites were accordingly annotated (putative identification).

MetaboAnalyst version 5.0 (https://www.metaboanalyst.ca/) accessed on 12 August 2021, a web-based tool for the analysis of metabolomics data sets, was used for further metabolic data analysis and functional interpretation for heat map visualisation and pathway mapping [84]. Peak intensities of metabolomic data were imported into MetaboAnalyst, transformed and Pareto-scaled to reduce systemic variance within the features. The average integrated peak intensities of the annotated metabolites were used to construct the heat maps with the statistical analysis software available on MetaboAnalyst (https://www.metaboanalyst.ca/) accessed on 12 August 2021. The group averages were used to show metabolite intensities from WT and mutants (*lbr2-2* and *bak1-4*) under different LPS chemotype treatments for all time points. Pathway analysis (PA) was performed by the MetPA (Metabolomics Pathway Analysis), a component of the MetaboAnalyst suite that uses the Kyoto Encyclopedia of Genes and Genomes (KEGG) metabolic pathways as the backend knowledge base. For the PA, KEGG IDs of the annotated metabolites were submitted to the MetaboAnalyst and significant mapped pathways were generated.

## 5. Conclusions

LPSs as a MAMP triggers defence responses in mammals and plants. The LPS perception and signalling in mammals are established, whereas in plants the PRR and/or co-receptor complex and the associated defence signalling are still unresolved. Understanding the metabolite profiles and perturbed metabolic pathways in WT Arabidopsis and mutants (*lbr2-2* and *bak1-4*) treated with LPSs will provide useful insight into the mechanism underlying plant defence responses against pathogens. The aim of the study was to investigate how the *lbr2-2* and *bak1-4* Arabidopsis mutants respond to LPS treatment in comparison to the WT, using untargeted LC-MS-based metabolomics. In this regard, two LPS chemotypes, originating from *P. syringae* and *X. campestris* (LPS*_Pst_* and LPS*_Xcc_*) that differ in the chemical ‘molecular patterns’ of the LPS sub-moieties, were applied. Both chemotypes were found to induce differential reprogramming of the metabolome in support of an enhanced defensive state in the WT, as well as in the *lbr2-2* and *bak1-4* mutants. Most of the annotated metabolites belong to the classes of ‘glucosinolates’, ‘benzoic–and HCA derivatives’, ‘flavonoids’, ‘lignans’, ‘lipids, oxylipin and arabidopsides’ and ‘phytohormones’. The presence and absence of certain metabolites as observed in the WT and mutants suggest that they responded differently to the two LPS chemotype treatments as a consequence of the functional LBR2 and BAK1 proteins in WT, but not in the respective mutants.

Furthermore, the Metabolomic Pathway Analysis (MetPA) of significantly annotated metabolites from WT, *lbr2-2* and *bak1-4* showed distinct altered pathways that include flavone and flavanol biosynthesis, phenylpropanoid biosynthesis, alpha-linolenic acid metabolism, and glucosinolate biosynthesis. The dissimilar metabolite profiles and metabolic responses (as seen in the heat maps) observed in WT and mutant plants after treatment with the two LPS chemotypes may be attributed to the distinct structures of the tripartite LPS chemotypes. In addition, the involvement of functional LBR2 and BAK1 proteins in the WT, but not in the respective mutants, could have contributed to the different metabolic responses of WT vs. *lbr2-2* vs. *bak1-4.* Moreover, the increased accumulation of defence-associated metabolites observed in the mutants indicates an inducible response to the LPS chemotypes (albeit different from that of the WT), indicating prior perception of the LPSs and subsequent signal processing. This might be attributable to a level of redundancy in LPS perception and point to the presence of other LPS-interacting/co-receptors that are participating/complementing the perception of intact LPS (or sub-moieties thereof) and resulting signalling in the mutants. Untargeted metabolomics was thus found to generate a wealth of information in monitoring plant biological systems such as profiling of differential metabolite accumulation as part of the adaptation of the host’s ‘defensome’ in response to pathogenic stresses, and these metabolites may thus serve as biomarkers in LPS:plant interaction studies.

## Figures and Tables

**Table 1:** Putatively annotated discriminatory metabolites, extracted from the OPLS-DA S-plots, for the Arabidopsis WT, *lbr2-2* and *bak1-4* leaves treated with LPS*_Pst_* and LPS*_Xcc_* chemotypes, and analysed in both ESI (+/−) MS modes. Metabolites that accumulated due to the specific LPS chemotype treatment after 0, 12, 18, and 24 h in WT, *lbr2-2* and *bak1-4*, respectively, are listed. Early and later time point LPS treatments are represented as 0–12 h and 18–24 h, respectively. Metabolites are coloured based for the plants they were identified in (only WT: green; WT and *lbr2-2*: pink; WT and *bak1-4*: yellow; only mutants: blue; all three lines: grey, and with a lighter shade for only one chemotype).

#	Annotated Metabolites	*m*/*z*	Rt (min)	Adducts	Molecular Formula	WT (h)		*lbr2-2* (h)		*bak1-4* (h)	
						*Pst*	*Xcc*	*Pst*	*Xcc*	*Pst*	*Xcc*
**Glucosinolates**											
1	8-(Methylsulphinyl)octyl cyanide (8-MeSO-octyl-CN)	202.126	13.45	[M + H]^+^	C_10_H_19_NOS	12, 18, 24	0, 12, 18, 24			24	12, 18,
2	8-(Methylsulphinyl)octyl isothiocyanate (Hirsutin)	234.096	18.48	[M + H]^+^	C_10_H_19_NOS_2_	0, 12, 18, 24	12, 18, 24	0, 12, 18, 24	0, 12, 18, 24		
3	7-Methylsulphinylheptyl isothiocyanate	220.080	17.13	[M + H]^+^	C_9_H_17_NOS_2_	12, 18, 24	0, 12	12	12, 24		12
4	8-(Methylsulphinyl)octylamine (8-MeSO-octyl-NH2)	192.141	2.41	[M + H]^+^	C_9_H_21_NOS	0, 12, 18, 24	0, 12, 18	12, 24	24		0, 12
5	4-Methylthiobutyl glucosinolate (Glucoerucin)	420.044	2.38	[ M−H ]^−^	C_12_H_23_NO_9_S_3_		12, 24				
6	3-Indolylmethyl glucosinolate (Glucobrassicin)	447.052	2.80	[ M−H ]^−^	C_16_H_19_N_2_O_9_S_2_		24				
7	8-Methylsulphinyloctyl glucosinolate (Glucohirsutin)	492.104	4.79	[ M−H ]^−^	C_16_H_31_NO_10_S_3_	12	24				
**Benzoic Acid and Hydroxycinnamic Acid Derivatives**											
8	6,7-Dimethoxycoumarin (scoparone)	207.066	11.50	[M + H]^+^	C_11_H_10_O_4_	0, 12, 18, 24	0, 12, 18, 24	0, 12, 18, 24	0, 12, 18, 24	0, 12, 18, 24	0, 18, 24
9	Sinapic acid	223.059	11.49	[ M−H ]^−^	C_11_H_12_O_5_	0, 12, 18, 24	0, 12, 18	0, 12, 18, 24	0, 12, 18, 24	0, 18	18
10	Sinapoyl malate	339.071	11.49	[ M−H ]^−^	C_15_H_16_O_9_	0, 18, 24	0, 12, 18	0, 12, 18, 24	0, 12, 18, 24	0, 12, 18, 24	0, 18, 24
11	2,5-Dihydroxybenzoic acid pentoside isomer I	285.059	3.24	[ M−H ]^−^	C_12_H_14_O_8_	0, 12, 18	18	0, 12, 18, 24	0, 12, 18, 24	12, 24	18, 24
12	2,5-Dihydroxybenzoic acid pentoside isomer II	285.060	4.53	[ M−H ]^−^	C_12_H_14_O_8_					0, 12	0, 12
13	1-O-Sinapoyl-beta-D-glucose	385.111	7.20	[ M−H ]^−^	C_17_H_22_O_10_	0	0, 12, 24			18	18
**Flavonoids**											
14	Afzelin (Kaempferol-3-rhamnoside)	433.108	12.71	[M + H]^+^	C_21_H_20_O_10_	24					
15	Robinin (Kaempferol-3-O-robinoside-7-O-rhamnoside	739.211	10.11	[ M−H ]^−^	C_33_H_40_O_19_	24				0	
16	Kaempferitrin (Kaempferol 3,7-dirhamnoside)	577.156	12.69	[ M−H ]^−^	C_27_H_30_O_14_	24	12			24	
17	Kaempferol 3-O-rhamnoside-7-O-glucoside	593.149	11.72	[ M−H ]^−^	C_27_H_30_O_15_	24	24				
18	2’,4’,4-Trihydroxy-3’-prenylchalcone	323.133	4.04	[ M−H ]^−^	C_20_H_20_O_4_	18					18
Lignans *											
19	G(8-O-4)G hexoside	537.196	5.30	[ M−H ]^−^	C_26_H_34_O_12_		24			18	
20	Lariciresinol hexoside	521.201	11.72	[ M−H ]^−^	C_26_H_34_O_11_	0, 12, 18, 24				0, 12, 18	0, 12
21	G(8–5)FA malate	487.128	14.55	[ M−H ]^−^	C_24_H_24_O_11_	0		0, 18, 24			
**Lipids, Oxylipins and Arabidopsides**											
22	Methyl 8-hydroxy-11E,17-Octadecadien-9-ynoate	307.223	23.55	[M + H]^+^	C_19_H_30_O_3_	12, 18, 24	0, 12, 18, 24	0, 12, 18, 24	0, 12, 18, 24	12, 18, 24	0, 12, 18, 24
23	9,12,13-Trihydroxy-10,15-octadecadienoic acid	327.216	17.10	[ M−H ]^−^	C_18_H_32_O_5_	0, 12, 18, 24	0, 12, 18, 24	0, 12, 18, 24	0, 12, 18, 24	12, 18, 24	0, 12, 18, 24
24	9,12,13-Trihydroxyoctadec-10-enoic acid (9, 12, 13-TriHOME)	329.232	17.75	[ M−H ]^−^	C_18_H_34_O_5_	12, 18, 24	0, 12, 18, 24	0, 12, 18, 24	0, 12, 18, 24	18, 24	18, 24
25	13S-Hydroperoxy-9Z, 11E, 15Z octadecatrienoic acid (13(S)-HPOTrE)	309.206	20.76	[ M−H ]^−^	C_18_H_30_O_4_	18, 24	0, 12, 18, 24	0, 18	0, 24	0	12, 18, 24
26	7S,8S-Dihydroxy-9Z,12Z-octadecadienoic acid (7S,8S-DiHODE)	311.221	20.34	[ M−H ]^−^	C_18_H_32_O_4_	12, 18, 24	0, 12, 18, 24	0, 18, 24	0, 12, 18, 24	24	24
27	Methyl 9,12-dihydroxy-13-oxo-10-octadecenoate	341.231	18.73	[ M−H ]^−^	C_19_H_34_O_5_	18	18				
28	3’-O-Linolenoylglyceryl 6-O-galactopyranosyl-galactopyranoside isomer I	721.366	20.96	[ M−H + FA]^−^	C_33_H_56_O_14_					0, 12	
29	3’-O-Linolenoylglyceryl 6-O-galactopyranosyl-galactopyranoside isomer II	721.365	21.21	[ M−H + FA]^−^	C_33_H_56_O_14_	0, 18		12, 18	0,12	0	
30	Arabidopside A	775.463	23.10	[M + H]^+^	C_43_H_66_O_12_	0, 12, 18, 24	12, 18, 24				
31	Arabidopside D	1,009.500	22.85	[ M−H + FA]^−^	C_51_H_80_O_17_	0, 12	12	0, 12			
32	12-Oxo-phytodienoic Acid (12-OPDA)	291.198	21.26	[ M−H ]^−^	C_18_ H_28_O_3_	18, 24	0, 12, 18, 24	0, 18, 24	0, 12, 18, 24	18, 24	12, 18, 24
33	Dinor-12-oxo phytodienoic acid (dinor-OPDA)	263.163	19.50	[ M−H ]^−^	C_16_H_24_O_3_	0, 12, 18, 24	0, 12, 18, 24	0, 12, 18, 24	0, 12, 18, 24	12, 18, 24	0, 12, 18, 24
34	*Sn*2-O-(dinoroxophytodienoyl)- monogalactosyl monogylceride	545.261	16.84	[ M−H + FA]^−^	C_25_H_40_O_10_	0, 12		0	0	0, 12	12
35	*Sn*2-O-(dinoroxophytodienoyl)-digalactosyl isomer I	707.317	15.96	[ M−H + FA]^−^	C_31_H_50_O_15_		18, 24	0, 18		12	12, 24
36	*Sn*2-O-(dinoroxophytodienoyl)-digalactosyl isomer II	707.312	16.31	[ M−H + FA]^−^	C_31_H_50_O_15_			0, 18, 24	18, 24	18	24
37	*Sn*1-O-(12-oxophytodienoyl)-digalactosyl monoglyceride isomer I	735.351	17.64	[ M−H + FA]^−^	C_33_H_54_O_15_	0, 12, 18	0, 12, 18, 24	0, 12		0, 12, 18	0, 12, 24
38	*Sn*1-O-(12-oxophytodienoyl)-digalactosyl monoglyceride isomer II	735.351	17.96	[ M−H + FA]^−^	C_33_H_54_O_15_	0, 24	12, 18				12, 24
**Phytohormones**											
39	Abscisic acid	265.177	19.51	[M + H]^+^	C_15_H_20_O_4_	18, 24	0, 12, 18, 24	0, 12, 18, 24	0, 12, 18, 24	18, 24	12, 18, 24
40	Salicylic acid 2-O-beta-D-glucoside	299.075	4.10	[ M−H ]^−^	C_13_H_16_O_8_					12	0, 12
**Others**											
41	L-Threonine	120.080	1.88	[M + H]^+^	C_4_H_9_NO_3_			12, 18	0, 12	12, 18, 24	0, 12, 18, 24
42	Citric acid	191.016	1.05	[ M−H ]^−^	C_6_H_8_O_7_	12, 18	18	12, 18, 24	12, 18, 24	0, 12, 18, 24	0, 12, 18, 24
43	Adenosine	268.104	1.17	[M + H]^+^	C_10_H_13_N_5_O_4_	0, 12, 18, 24	0, 12, 18, 24	0, 12, 18, 24	0, 12, 18, 24	0, 12, 18, 24	0, 12, 18, 24
44	Corchoionoside C	431.189	8.54	[ M−H + FA]^−^	C_19_H_30_O_8_	0, 12, 18	0, 12	0, 18	0, 18, 24	12, 18	0, 12
45	Sulforaphane-glutathione	485.116	2.86	[M + H]^+^	C_16_H_28_N_4_O_7_S_3_			0, 12, 18, 24	12, 18, 24		

* Shorthand naming of lignans nomenclature, as introduced by Morreel et al. [27].

## Data Availability

The information regarding the study design, LC-MS data, data processing and analyses are reported on and incorporated into the main text. Raw data, analyses and data processing information, and the meta-data are deposited to the EMBL-EBI metabolomics repository—MetaboLights50, with the identifier MTBLS4468 (www.ebi.ac.uk/metabolights/MTBLS4468) accessed on 15 March 2022.

## Supplementary Materials

The following supporting information can be downloaded at: https://www.mdpi.com/article/10.3390/metabo12050379/s1. Figure S1: Representative UHPLC-MS BPI chromatograms of methanolic extracts from WT Arabidopsis in ESI (−) data mode. Figure S2: Unsupervised chemometric modelling of LC-MS-analysed Arabidopsis WT leaf extracts (ESI (−) data). Figure S3: OPLS-DA modelling of Arabidopsis WT leaf extracts (ESI (+) data). Figure S4: OPLS-DA modelling of Arabidopsis WT leaf extracts (ESI (+) data). Figure S5: A representative receiver operator characteristic (ROC) plot summarising the performance of binary classifiers (OPLS-DA). Figure 6: Heatmap presentation of significant annotated metabolites in ESI (+) mode. Figure S7: Alpha-linolenic acid metabolism analysis by Metabolomic Pathway Analysis (MetPA). Figure S8: Phenylpropanoid biosynthesis analysis by Metabolomic Pathway Analysis (MetPA). Figure S9: Flavone and flavonol biosynthesis analysis by Metabolomic Pathway Analysis (MetPA). Figure S10: Glucosinolate biosynthesis analysis by Metabolomic Pathway Analysis (MetPA). Table S1: Diagnostic fragments of annotated metabolites and KEGG IDs in Table 1. Table S2: List of significant metabolic pathways that were altered by the treatment of Arabidopsis WT with LPS*_Pst_* and LPS*_Xcc_* as generated by Metabolomic Pathway Analysis (MetPA). Table S3: List of significant metabolic pathways that were altered by the treatment of Arabidopsis *lbr2-2* mutant with LPS*_Pst_* and LPS*_Xcc_* as generated by Metabolomic Pathway Analysis (MetPA). Table S4: List of significant metabolic pathways that were altered by the treatment of Arabidopsis *bak1-4* mutant with LPS*_Pst_* and LPS*_Xcc_* as generated by Metabolomic Pathway Analysis (MetPA).
