# Supplementary material for: Untargeted Metabolomics Profiling of Arabidopsis WT, lbr-2-2 and bak1-4 Mutants Following Treatment with Two LPS Chemotypes
Source: Metabolites. 2022 Apr 22;12(5):379. doi: 10.3390/metabo12050379 (PMC9146344; doi:10.3390/metabo12050379)
Supplement: Supplementary file 1 [file metabolites-12-00379-s001.zip › metabolites-1680136-supplementary.pdf]

Untargeted metabolomics profiling of Arabidopsis WT, *lbr-2-2* and *bak1-4* mutants following treatment with two LPS chemotypes

Benedict C. Offor, Msizi I. Mhlongo, Paul A. Steenkamp, Ian A. Dubery and Lizelle A. Piater *

Department of Biochemistry, University of Johannesburg, Auckland Park, 2006, South Africa;

benedictoffor@gmail.com (B.C.O.); mmhlongo@uj.ac.za (M.I.M); psteenkamp@uj.ac.za (P.A.S.); idubery@uj.ac.za (I.A.D.)

* Correspondence: lpiater@uj.ac.za (L.A.P); Tel.: +27-11-559-2403

Supplementary materials

The aims and objectives of the study, as well as the study design and experimental data files were deposited in the MetaboLights data depository (Accession number MTBLS4468). All relevant Figures and Tables refered to but not included in the main text are provided in this supplementary file. Other figures (including some ESI (+) data and *lbr2-2* and *bak1-4* mutant plants data generated by multivariate data analysis) and all experimental raw data are available on request from Prof. L.A. Piater, Department of Biochemistry, University of Johannesburg.

Ultra-high performance liquid-chromatography-mass spectometry chromatograms (ESI (–) data)

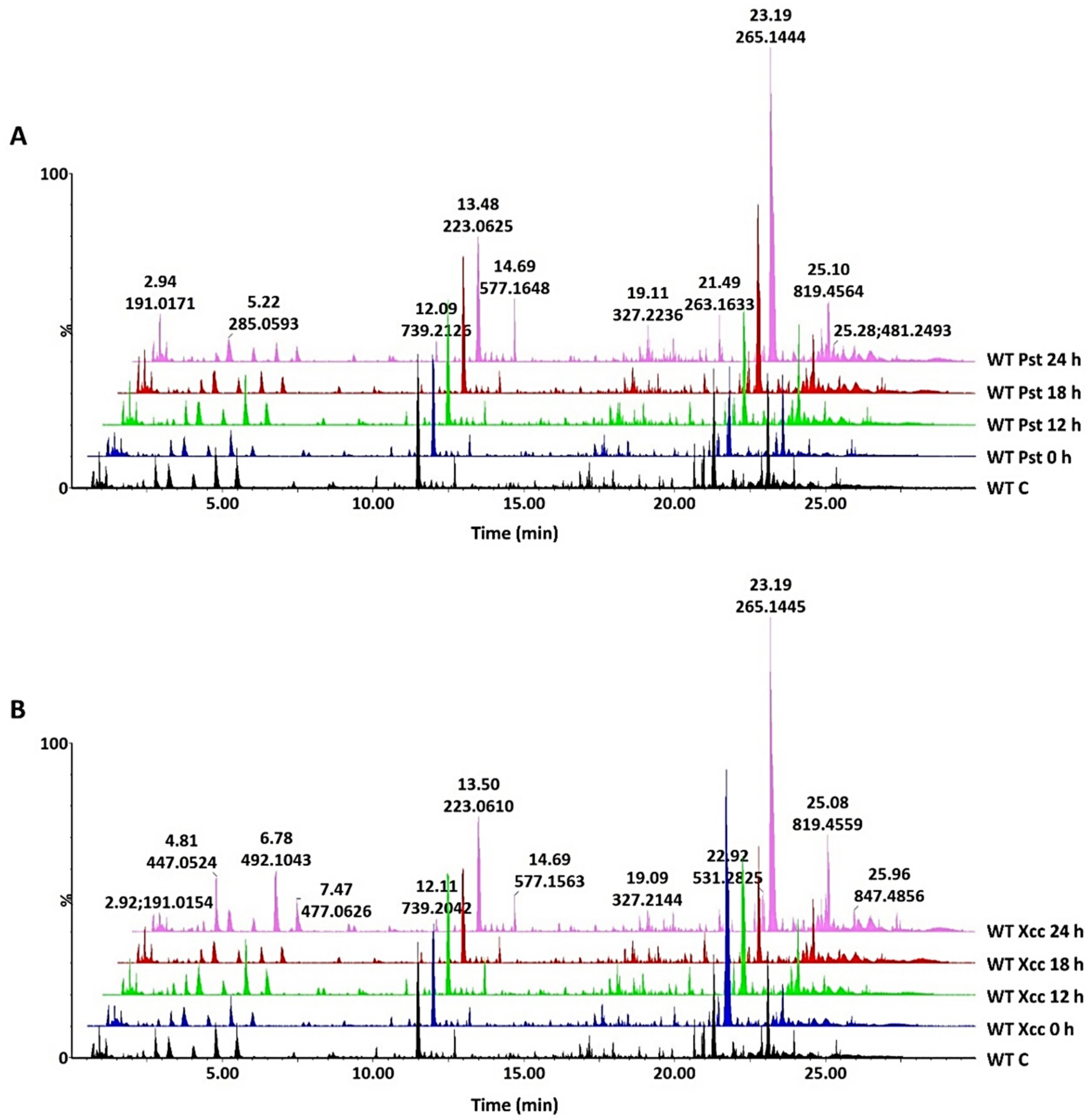

Figure S1. Representative UHPLC-MS BPI chromatograms of methanolic extracts from WT Arabidopsis in ESI (–) data mode. **(A)** Extracts from LPS*Pst*-treated leaves. **(B)** Extracts from LPS*Xcc*-treated leaves. In both **A** and **B**, chromatograms are overlayed as control, 0, 12, 18 and 24 h post-LPS treatment. Observed time-related presence/absence of peaks and differential peak intensities indicates metabolomic changes as a result of the LPS treatment.

Unsupervised chemometric modelling of (ESI (–) data)

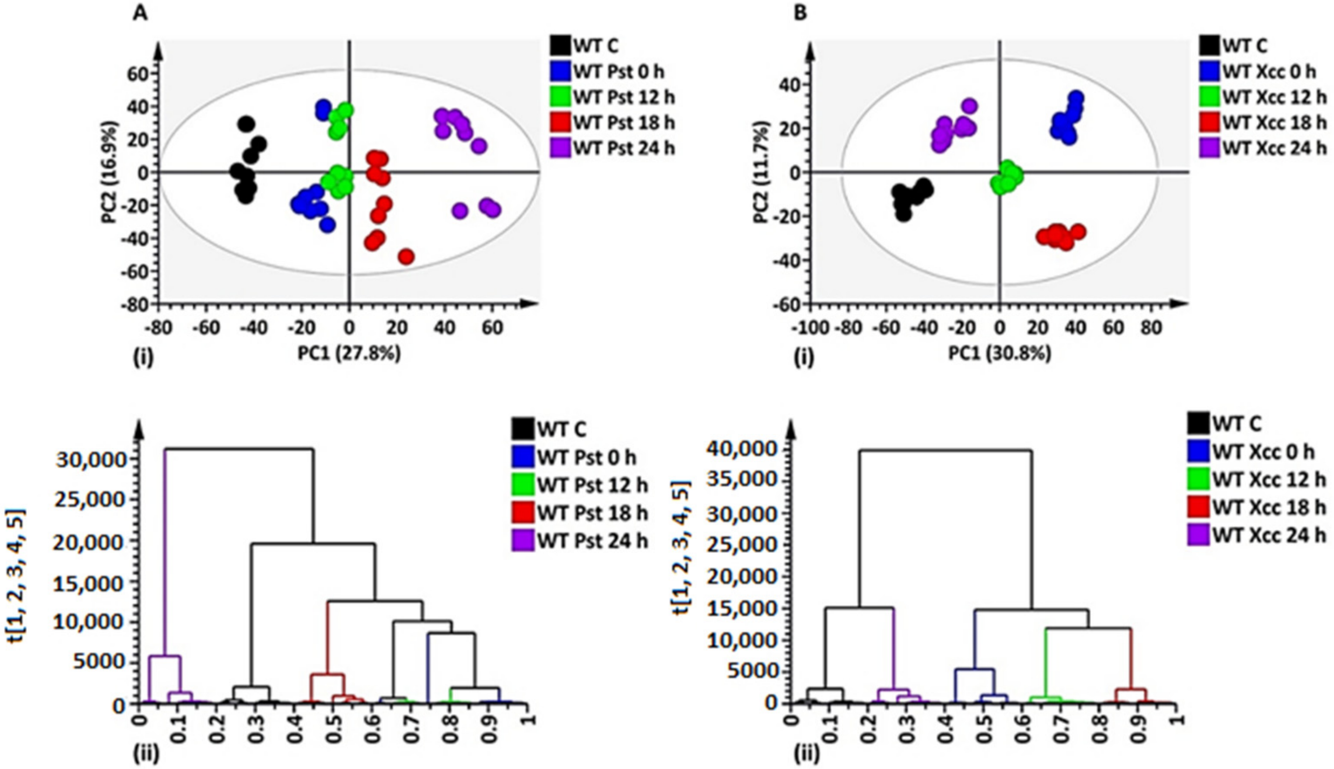

Figure S2. Unsupervised chemometric modelling of LC-MS-analysed Arabidopsis WT leaf extracts (ESI (–) data). **A**,**B** **(i)** represent principal component analysis (PCA) score plots of control *vs*. 0, 12, 18 and 24 h LPS*Pst* - and LPS*Xcc*-treated WT, respectively. **A**,**B** **(ii)** represent hierarchical clustering analysis (HiCA) dendrogram of control *vs*. 0, 12, 18 and 24 h LPS*Pst* - and LPS*Xcc*-treated WT, respectively. Model parameters are: **(A)** **(i)** $R^2X = 65.7\%/Q^2 = 46.4\%$, **(B)** **(i)** $R^2X = 65.6\%/Q^2 = 47.1\%$. PCA and (HiCA) dendrogram are coloured according to different time treatments.

Orthogonal projection to latent structures discriminant analysis (OPLS-DA) models of (ESI (+) data)

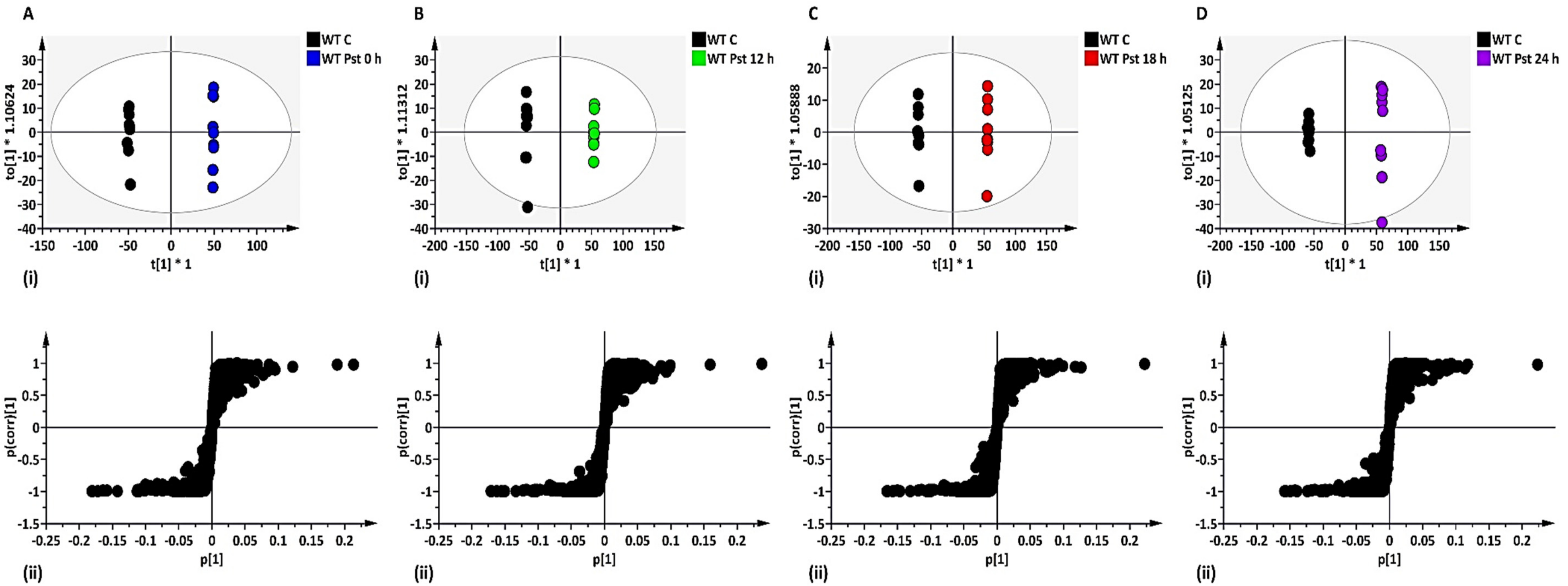

Figure S3. OPLS-DA modelling of Arabidopsis WT leaf extracts (ESI (+) data). **A**-**D** **(i)** represent OPLS-DA score plots showing clear separation between control *vs*. LPS_{Pst} treatment after 0, 12, 18, and 24 h, respectively. **A**-**D** **(ii)** represent OPLS-DA loading S-plots showing the discriminant features (ions) responsible for the sample grouping observed in **A**-**D** **(i)**. The OPLS-DA model parameters were: **(A)** R^2X = 72.5%/Q^2 = 99.8%, **(B)** R^2X = 76.3%/Q^2 = 99.8%, **(C)** R^2X = 78.3%/Q^2 = 99.9%, **(D)** R^2X = 79.9%/Q^2 = 99.9%, respectively. The variables in the top right quadrants of the S-plots correlated positively to the treatment. Selected discriminant ions for downstream metabolite identifications were based of a correlation [p(corr)] of ≥ 0.5 and covariance of (p1) ≥ 0.05.

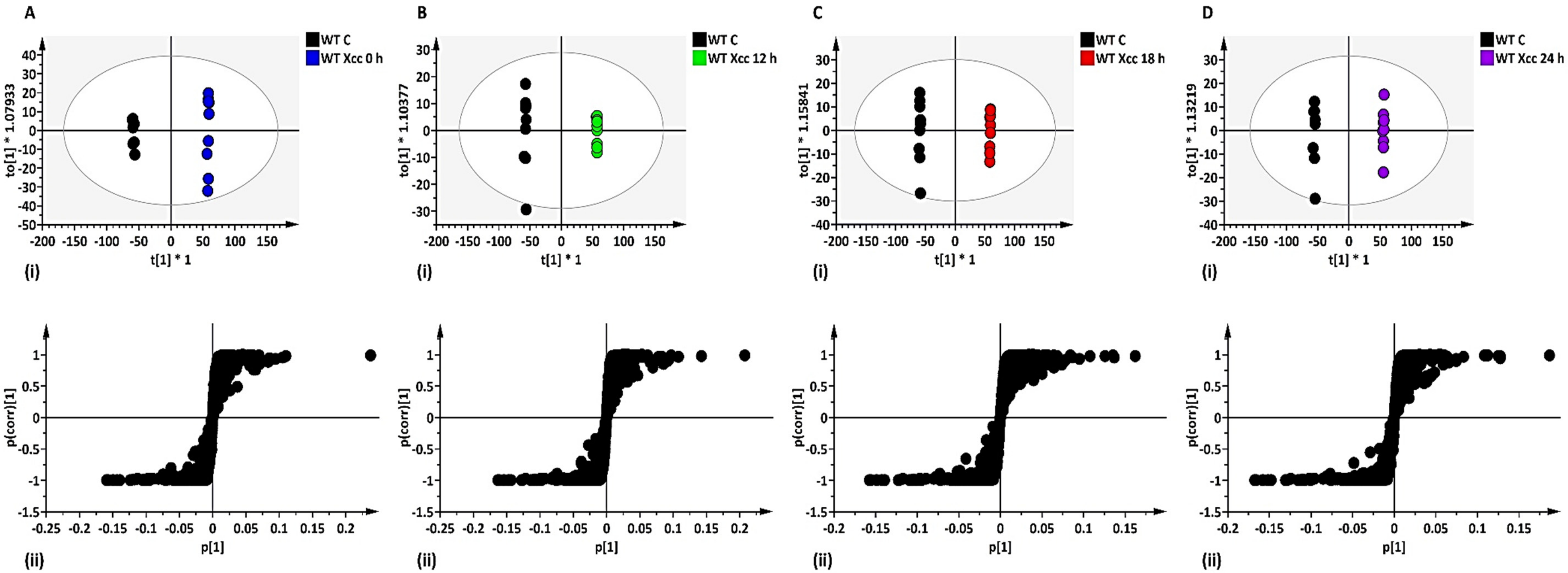

Figure S4. OPLS-DA modelling of Arabidopsis WT leaf extracts (ESI (+) data). **A**-**D** **(i)** represent OPLS-DA score plots showing clear separation between control *vs.* LPS_{Xcc} treatment after 0, 12, 18, and 24 h, respectively. **A**-**D** **(ii)** represent OPLS-DA loading S-plots showing the discriminant features (ions) responsible for the sample grouping observed in **A**-**D** **(i)**. The OPLS-DA model parameters were: **(A)** R^2X = 78.6%/Q^2 = 99.9%, **(B)** R^2X = 78.5%/Q^2 = 99.9%, **(C)** R^2X = 79.9%/Q^2 = 99.9%, **(D)** R^2X = 78.5%/Q^2 = 99.9%, respectively. The variables in the top right quadrants of the S-plots correlated positively to the treatment. Selected discriminant ions for downstream metabolite identifications were based of a correlation [p(corr)] of ≥ 0.5 and covariance of (p1) ≥ 0.05.

Receiver operator characteristic (ROC) plots

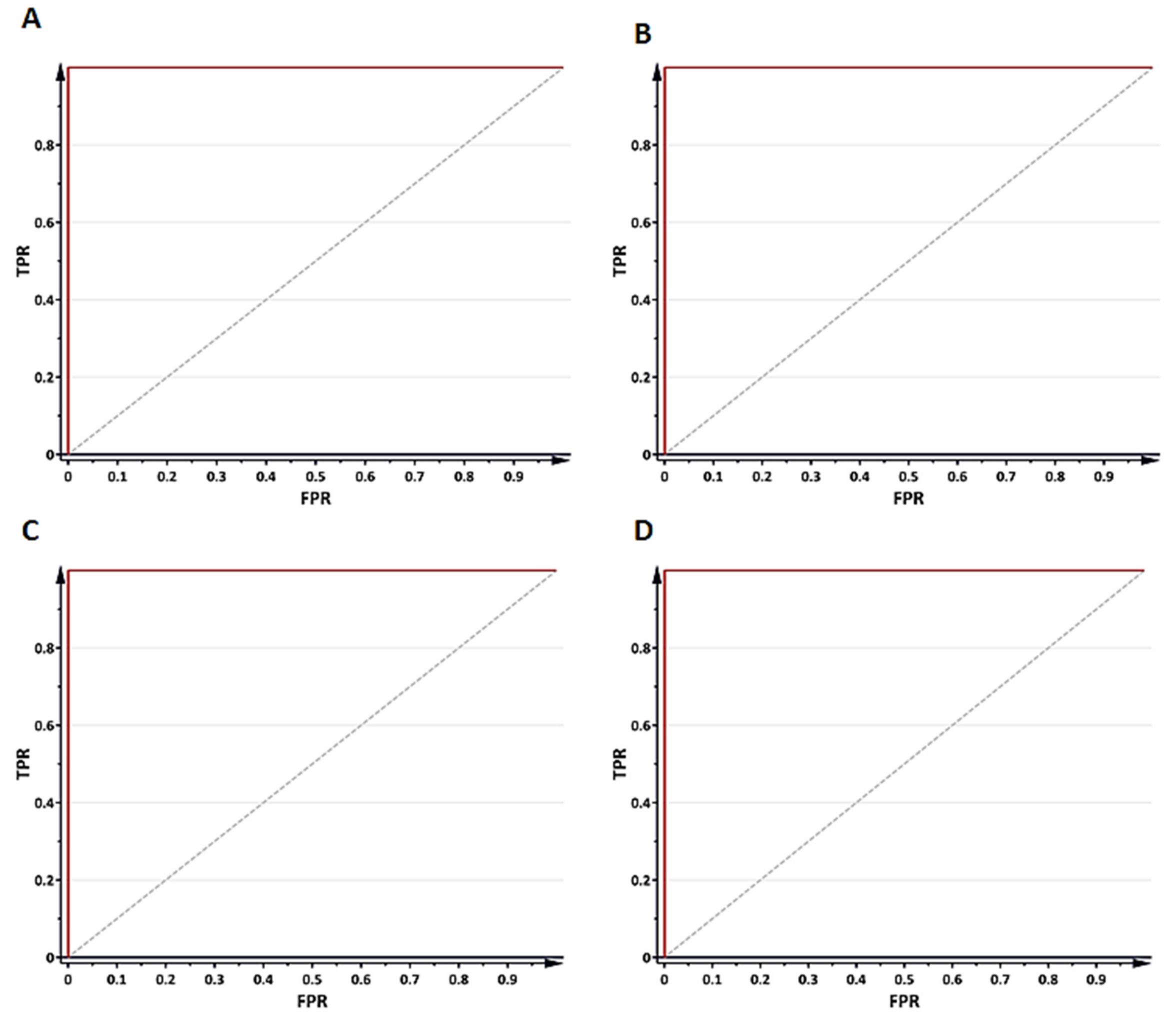

Figure S5. A representative receiver operator characteristic (ROC) plot summarising the performance of binary classifiers (OPLS-DA). **A**, **B**, **C**, **D** represents ROC plots for control *vs*. 0, 12, 18, and 24 h LPS$_{Pst}$-treated Arabidopsis WT, respectively. The plot shows that the computed OPLS-DA models are excellent classifiers with 100% sensitivity and 100% specificity as depicted by the ROC curve that passes through the top-left corner.

Diagnostic fragmentation patterns and KEGG identifiers of annotated metabolites

Table S1. Diagnostic fragments of annotated metabolites and KEGG IDs in **Table 1**.

Annotated metabolites	*m/z*	Rt (min)	Adducts	Molecular formula	Fragmentation ions	KEGG ID
L-Threonine	120.08	1.88	$[M+H]^+$	$C_4H_9NO_3$	103, 93, 91, 77	C00188
Citric acid	191.016	1.05	$[M-H]^-$	$C_6H_8O_7$	173, 111	C00158
Afzelin (Kaempferol-3-rhamnoside)	433.108	12.71	$[M+H]^+$	$C_{21}H_{20}O_{10}$	287	C16911
Robinin (Kaempferol-3-O-robinoside-7-O-rhamnoside	739.211	10.11	$[M-H]^-$	$C_{33}H_{40}O_{19}$	593, 430, 284	C10178
Kaempferitrin (Kaempferol 3,7-dirhamnoside)	577.156	12.69	$[M-H]^-$	$C_{27}H_{30}O_{14}$	431, 285	C16981
Kaempferol 3-O-rhamnoside-7-O-glucoside	593.149	11.72	$[M-H]^-$	$C_{27}H_{30}O_{15}$	447, 430, 285	C21854
2',4',4-Trihydroxy-3'-prenylchalcone	323.133	4.04	$[M-H]^-$	$C_{20}H_{20}O_4$	119, 101	
8-(Methylsulphinyl)octyl cyanide (8-MeSO-octyl-CN)	202.126	13.45	$[M+H]^+$	$C_{10}H_{19}NOS$	138, 121, 109, 96, 93, 82, 79	
8-(Methylsulphinyl)octyl isothiocyanate (Hirsutin)	234.096	18.48	$[M+H]^+$	$C_{10}H_{19}NOS_2$	170, 137	
7-Methylsulphinylheptyl isothiocyanate	220.08	17.13	$[M+H]^+$	$C_9H_{17}NOS_2$	156, 123	
8-(Methylsulphinyl)octylamine (8-MeSO-octyl-NH2)	192.141	2.41	$[M+H]^+$	$C_9H_{21}NOS$	175, 128, 111, 69	
4-Methylthiobutyl glucosinolate (Glucoerucin)	420.044	2.38	$[M-H]^-$	$C_{12}H_{23}NO_9S_3$	260, 160, 96, 74	C08409
3-Indolylmethyl glucosinolate (Glucobrassicin)	447.052	2.8	$[M-H]^-$	$C_{16}H_{19}N_2O_9S_2$	306, 259, 96	C05837
8-Methylsulfinyloctyl glucosinolate (Glucohirsutin)	492.104	4.79	$[M-H]^-$	$C_{16}H_{31}NO_{10}S_3$	428, 259, 234, 96	C17271
6,7-Dimethoxycoumarin (scoparone)	207.066	11.5	$[M+H]^+$	$C_{11}H_{10}O_4$	175, 147, 119, 91	C09311
Sinapic acid	223.059	11.49	$[M-H]^-$	$C_{11}H_{12}O_5$	208, 193, 179, 164, 149, 121	C00482
Sinapoyl malate	339.071	11.49	$[M-H]^-$	$C_{15}H_{16}O_9$	223, 208, 164, 149, 121	C02887
2,5-Dihydroxybenzoic acid pentoside Isomer I	285.059	3.24	$[M-H]^-$	$C_{12}H_{14}O_8$	153, 152, 108	

2,5-Dihydroxybenzoic acid pentoside isomer II	285.06	4.53	$[M-H]^-$	$C_{12}H_{14}O_8$	153, 152, 109	
1-O-Sinapoyl-beta-D-glucose	385.111	7.2	$[M-H]^-$	$C_{17}H_{22}O_{10}$	307, 223, 205, 190	C01175
G(8-O-4)G hexoside	537.196	5.3	$[M-H]^-$	$C_{26}H_{34}O_{12}$	375, 345, 327, 297	
Lariciresinol hexoside	521.201	11.72	$[M-H]^-$	$C_{26}H_{34}O_{11}$	359, 329	
G(8–5)FA malate	487.128	14.55	$[M-H]^-$	$C_{24}H_{24}O_{11}$	371, 353, 341, 338, 294	
Methyl 8-hydroxy-11E,17-octadecadien-9-ynoate	307.223	23.55	$[M+H]^+$	$C_{19}H_{30}O_3$	275, 263, 257, 239, 219, 207	
9,12,13-Trihydroxy-10,15-octadecadienoic acid	327.216	17.1	$[M-H]^-$	$C_{18}H_{32}O_5$	309, 229, 211, 183, 171	
9,12,13-Trihydroxyoctadec-10-enoic acid (9, 12, 13-TriHOME)	329.232	17.75	$[M-H]^-$	$C_{18}H_{34}O_5$	291, 229, 211, 197, 171	C14833
13S-Hydroperoxy-9Z, 11E, 15Z-octadecatrienoic acid (13(S)-HPOTrE)	309.206	20.76	$[M-H]^-$	$C_{18}H_{30}O_4$	291, 277, 177, 153	C04785
7S,8S-Dihydroxy-9Z,12Z-octadecadienoic acid (7S,8S-DiHODE)	311.221	20.34	$[M-H]^-$	$C_{18}H_{32}O_4$	309, 291, 263, 253, 197, 171	C07354
Methyl 9,12-dihydroxy-13-oxo-10-octadecenoate	341.231	18.73	$[M-H]^-$	$C_{19}H_{34}O_5$	283, 263, 249, 225, 171	
3'-O-Linolenoylglyceryl 6-O-galactopyranosyl-galactopyranoside isomer I	721.366	20.96	$[M-H+FA]^-$	$C_{33}H_{56}O_{14}$	675, 415, 397, 277	
3'-O-Linolenoylglyceryl 6-O-galactopyranosyl-galactopyranoside isomer II	721.365	21.21	$[M-H+FA]^-$	$C_{33}H_{56}O_{14}$	675, 415, 397, 277	
Adenosine	268.104	1.17	$[M+H]^+$	$C_{10}H_{13}N_5O_4$	136	C00212
Sulforaphane-glutathione	485.116	2.86	$[M+H]^+$	$C_{16}H_{28}N_4O_7S_3$	472, 410, 356, 207, 136	
Arabidopside A	775.463	23.1	$[M+H]^+$	$C_{43}H_{66}O_{12}$	752, 729, 713, 613, 595, 349, 321, 275, 177, 133	
Arabidopside D	1009.5	22.85	$[M-H+FA]^-$	$C_{51}H_{80}O_{17}$	963, 791, 671, 481, 397, 311, 291, 277	
12-Oxo-phytodienoic acid (12-OPDA)	291.198	21.26	$[M-H]^-$	$C_{18}\ H_{28}O_3$	277, 273, 265, 247, 96	C01226
Dinor-12-oxo-phytodienoic acid (dinor-OPDA)	263.163	19.5	$[M-H]^-$	$C_{16}H_{24}O_3$	245, 237, 219, 191, 165, 96	
*Sn*2-O-(dinoroxophytodienoyl)- monogalactosyl monoglyceride	545.261	16.84	$[M-H+FA]^-$	$C_{25}H_{40}O_{10}$	499, 263, 253, 245	

*Sn*2-O-(dinoroxophytodienoyl)-digalactosyl isomer I	707.317	15.96	[M-H+FA]-	$C_{31}H_{50}O_{15}$	661, 415, 397, 384, 308, 263	
*Sn*2-O-(dinoroxophytodienoyl)-digalactosyl isomer II	707.312	16.31	[M-H+FA]-	$C_{31}H_{50}O_{15}$	661, 415, 397, 384, 263	
*Sn*1-O-(12-oxophytodienoyl)-digalactosyl monoglyceride isomer I	735.351	17.64	[M-H+FA]-	$C_{33}H_{54}O_{15}$	689, 414, 397, 291	
*Sn*1-O-(12-oxophytodienoyl)-digalactosyl monoglyceride isomer II	735.351	17.96	[M-H+FA]-	$C_{33}H_{54}O_{15}$	689, 414, 397, 291	
Abscisic acid	265.177	19.51	[M+H]+	$C_{15}H_{20}O_4$	247, 229, 219, 135	C06082
Salicylic acid 2-O-beta-D-glucoside	299.075	4.1	[M-H]-	$C_{13}H_{16}O_8$	137	
Corchoionoside C	431.189	8.54	[M-H+FA]-	$C_{19}H_{30}O_8$	385, 255, 223, 205, 153	

Glucosinolates such as glucoerucin and glucobrassicin were identified only in LPS_{Xcc}-treated WT while glucohirsutin was identified in both LPS chemotypes-treated WT. On the other hand, glucosinolate degradation products were identified in both WT and mutants. For instance, 8-MeSO-octyl-CN was identified in both LPS chemotypes-treated WT and *bak1-4*, but not in *lbr2-2.* Another glucosinolate degradation product, hirsutin, was identified in both LPS chemotypes-treated WT and *lbr2-2* but not in *bak1-4*. Overall, glucosinolates accumulated more in LPS_{Xcc}-treated plants compared to treatment with LPS_{Pst}. In addition, LPS induced accumulation of most glucosinolates in the WT followed by *lbr2-2* and then lastly *bak1-4*.

While selected benzoic – and HCA derivatives including sinapic acid and sinapoyl malate were identified in all plant lines, 1-O-sinapoyl-beta-D-glucose was identified only in WT and *bak1-4* mutant treated with both LPS chemotypes. Furthermore, 2,5-dihydroxybenzoic acid pentoside isomer I was identified in all lines whereas 2,5-dihydroxybenzoic acid pentoside isomer II was identified only in *bak1-4*, and in re-sponse to both LPS chemotypes. Overall, LPS_{Xcc}-treated plants showed slightly more accumulation of benzoic- and HCA derivatives compared to treatment with LPS_{Pst}. LPS-induced benzoic- and HCA derivatives were accumulated slightly more in *bak1-4* followed by *lbr2-2* and lastly by WT.

Although flavonoids were not identified as discriminant markers in LPS-treated *lbr2-2*, they were mostly accumulated at later time points in WT followed by *bak1-4*. Of special interest is the number of derivatives of the tetrahydroxyflavone, kaempferol. Here, afzelin (kaempferin / kaempferol 3-rhamnoside) was identified only at LPS_{Pst}-treated WT, where-as kaempferitrin (kaempferol 3,7-di-O-alpha-L-rhamnoside) was identified in both LPS chemotypes-treated WT and LPS_{Pst}-treated *bak1-4*. In addition, robinin (kaempferol-3-O-galactosyl-rhamnosyl-7-O-rhamnoside) was identified only in LPSPst-treated WT and kaempferol 3-O-rhamnoside-7-O-glucoside was identified in WT treated with both LPS chemotypes. Overall, LPS_{Pst} triggered the accumulation of more fla-vonoids compared to when treated with LPS_{Xcc}.

Lignans were identified in both WT and mutants. LPS_{Pst} induced accumulation of slightly more lignan in *bak1-4* followed by WT and lastly *lbr2-2*. LPS_{Xcc} did not trigger identification of lignan in the *lbr2-2* mutant, nevertheless, it induced identification of G(8-O-4)G hexoside and lariciresinol hexoside in WT and *bak1-4*, respectively.

Most of the 'lipids, oxylipin and arabidopsides' metabolites including methyl 8-hydroxy-11E,17-octadecadien-9-ynoate, 9,12,13-trihydroxy-10,15-octadecadienoic acid, 9, 12, 13-triHOME, 13(S)-HPOTrE, 7S,8S-DiHODE, 12-OPDA and dinor-OPDA were identified in all lines treated with both LPS chemotypes. Some exceptions include methyl 9,12-dihydroxy-13-oxo-10-octadecenoate, arabidopside A identified in only both LPS chemotypes-treated WT and arabidopside D identified in WT and *lbr2-2* only. In all, LPS induced accumulation of most 'lipids, oxylipin and arabidopsides' in the *lbr2-2* followed by *bak1-4* and lastly WT. While the phytohormone ABA was identified in all lines, salicyl-ic acid 2-O-beta-D-glucoside was identified only in both LPS chemotypes-treated *bak1-4*.

In summary, more accumulation of glucosinolates was observed in LPS$_{Xcc}$-treated plants than in the LPS$_{Pst}$ treatments; also WT plants accumulated most of these metabolites followed by the *lbr2-2* and then *bak1-4*. Benzoic- and HCA derivatives accumulated more in LPS$_{Xcc}$-treated plants than the LPS$_{Pst}$ treatment, with slightly more accumulation observed in *bak1-4* followed by *lbr2-2* and then WT. While flavonoids accumulated more in LPS$_{Pst}$-treated plants than those treated with LPS$_{Xcc}$, there was more accumulation of this metabolite class in WT followed by *bak1-4*, with none observed in *lbr2-2*. Lignan accumulated mostly in the LPS$_{Pst}$-treated *bak1-4* followed by WT and then *lbr2-2*. Both LPS chemotypes induced accumulation of most 'lipids, oxylipin and arabidopsides' in the *lbr2-2* followed by *bak1-4* and WT.

*Heatmap analysis of metabolites in **ESI (+)** mode*

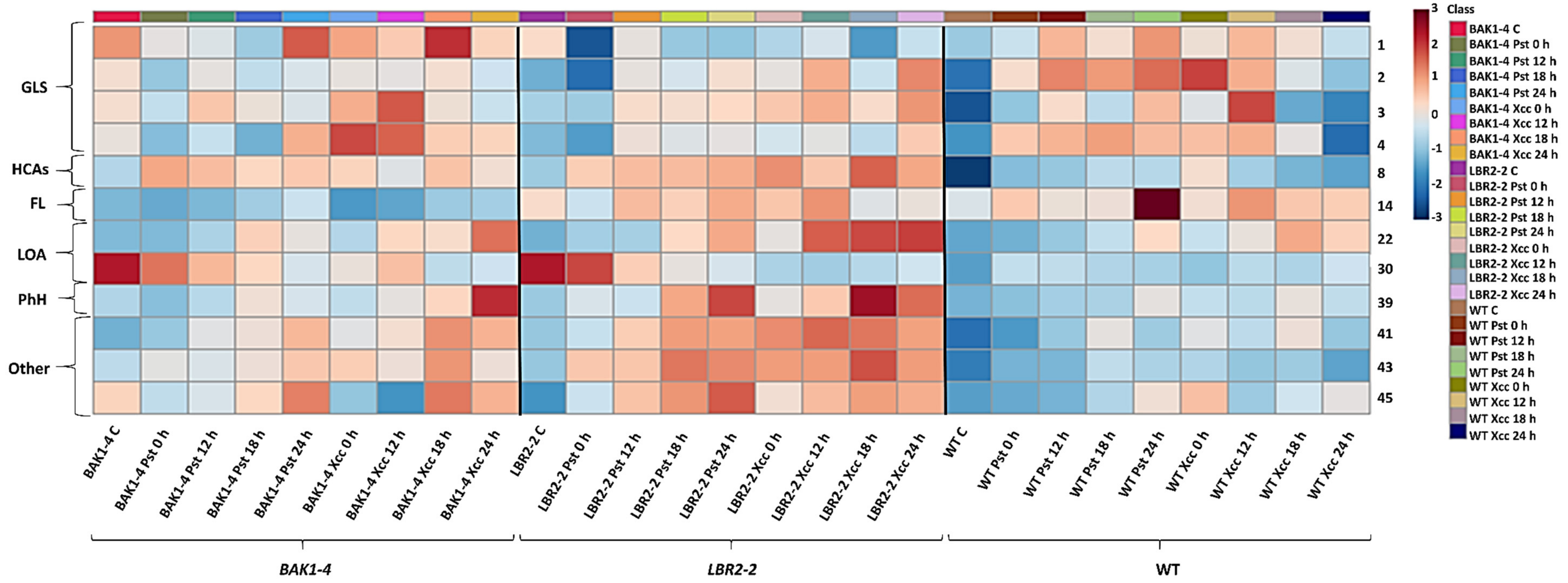

Figure S6. Heatmap presentation of significant annotated metabolites in **ESI (+)** mode. The LPS$_{Pst}$ - and LPS$_{Xcc}$ -induced annotated metabolites data of WT, *lbr2-2* and *bak1-4* were submitted to MetaboAnalyst and the relative intensities show the extent of metabolites accumulation. The rows represent the group of the identified metabolites (numbered as in **Table 1**) while the columns represented Arabidopsis plants with their respective LPS chemotype time-related treatments. The colour gradient of dark blue indicates lowest intensity while deep red indicates highest intensity. Metabolites are numbered as in **Table 1**.

Abbreviations: GLS = glucosinolates, HCAs = hydroxycinnamic acid derivatives, FL = flavonoids, LOA = lipids, oxylipins and arabidopsides, PhH = phytohormones.

List of significant metabolic pathways associated with changes to the Arabidopsis metabolomes in response to the LPS treatments

Table S2. List of significant metabolic pathways that were altered by the treatment of Arabidopsis WT with LPS_{Pst} and LPS_{Xcc} as generated by Metabolomic Pathway Analysis (MetPA). The 'Match status' indicates the number of compound hits per number of compounds that are in the particular pathway. The '*p*' represents *p*-value calculated from the enrichment analysis, '-log(*p*)' indicates negative logarithm of *p*-value, while 'Holm *p*' represents the *p*-value that was adjusted from the Holm-Bonferroni method. The 'impact' represents the impact of the pathways as calculated from the pathway topology analysis.

No.	Pathway name	Match status	*p*	-log(*p*)	Holm *p*	Impact
**1**	Alpha-linolenic acid metabolism	2/28	0.023587	1.6273	1.0	0.27666
**2**	Phenylpropanoid biosynthesis	2/46	0.059089	1.2285	1.0	0.0416
**3**	Flavone and flavonol biosynthesis	1/10	0.084529	1.073	1.0	0.0
**4**	Glucosinolate biosynthesis	2/65	0.10815	0.96598	1.0	0.0
**5**	Citrate cycle (TCA cycle)	1/20	0.16242	0.78937	1.0	0.11571
**6**	Tryptophan metabolism	1/28	0.22027	0.65704	1.0	0.0787
**7**	Glyoxylate and dicarboxylate metabolism	1/29	0.22724	0.64351	1.0	0.00702
**8**	Carotenoid biosynthesis	1/43	0.31893	0.4963	1.0	0.00632
**9**	Purine metabolism	1/63	0.43259	0.36392	1.0	0.00126

Table S3. List of significant metabolic pathways that were altered by the treatment of Arabidopsis *lbr2-2* mutant with LPS_{Pst} and LPS_{Xcc} as generated by Metabolomic Pathway Analysis (MetPA). The 'Match status' indicates the number of compound hits per number of compounds that are in the particular pathway. The '*p*' represents *p*-value calculated from the enrichment analysis, '-log(*p*)' indicates negative logarithm of *p*-value, while 'Holm *p*' represents the *p*-value that was adjusted from the Holm-Bonferroni method. The 'impact' represents the impact of the pathways as calculated from the pathway topology analysis.

No.	Pathway name	Match status	*p*	-log(*p*)	Holm *p*	Impact
**1**	Alpha-linolenic acid metabolism	2/28	0.017024	1.7689	1.0	0.27666
**2**	Phenylpropanoid biosynthesis	2/46	0.043331	1.3632	1.0	0.0416
**3**	Citrate cycle (TCA cycle)	1/20	0.13918	0.85643	1.0	0.11571
**4**	Valine, leucine and isoleucine biosynthesis	1/22	0.15208	0.81793	1.0	0.0
**5**	Glyoxylate and dicarboxylate metabolism	1/29	0.19586	0.70806	1.0	0.00702
**6**	Glycine, serine and threonine metabolism	1/33	0.21994	0.65769	1.0	0.1204
**7**	Carotenoid biosynthesis	1/43	0.27731	0.55703	1.0	0.00632
**8**	Aminoacyl-tRNA biosynthesis	1/46	0.29376	0.53201	1.0	0.0
**9**	Purine metabolism	1/63	0.3807	0.41942	1.0	0.00126

Table S4. List of significant metabolic pathways that were altered by the treatment of Arabidopsis *bak1-4* mutant with LPS_{Pst} and LPS_{Xcc} as generated by Metabolomic Pathway Analysis (MetPA). The 'Match status' indicates the number of compound hits per number of compounds that are in the particular pathway. The '*p*' represents *p*-value calculated from the enrichment analysis, '-log(*p*)' indicates negative logarithm of *p*-value, while 'Holm *p*' represents the *p*-value that was adjusted from the Holm-Bonferroni method. The 'impact' represents the impact of the pathways as calculated from the pathway topology analysis.

**No.**	**Pathway name**	**Match status**	***p***	**-log(*p*)**	**Holm *p***	**Impact**
**1**	Alpha-linolenic acid metabolism	2/28	0.017024	1.7689	1.0	0.27666
**2**	Phenylpropanoid biosynthesis	2/46	0.043331	1.3632	1.0	0.0416
**3**	Citrate cycle (TCA cycle)	1/20	0.13918	0.85643	1.0	0.11571
**4**	Valine, leucine and isoleucine biosynthesis	1/22	0.15208	0.81793	1.0	0.0
**5**	Glyoxylate and dicarboxylate metabolism	1/29	0.19586	0.70806	1.0	0.00702
**6**	Glycine, serine and threonine metabolism	1/33	0.21994	0.65769	1.0	0.1204
**7**	Carotenoid biosynthesis	1/43	0.27731	0.55703	1.0	0.00632
**8**	Aminoacyl-tRNA biosynthesis	1/46	0.29376	0.53201	1.0	0.0
**9**	Purine metabolism	1/63	0.3807	0.41942	1.0	0.00126

Comparative peak intensities of selected metabolites associated with identified metabolic pathways associated with the host response to the LPS treatments

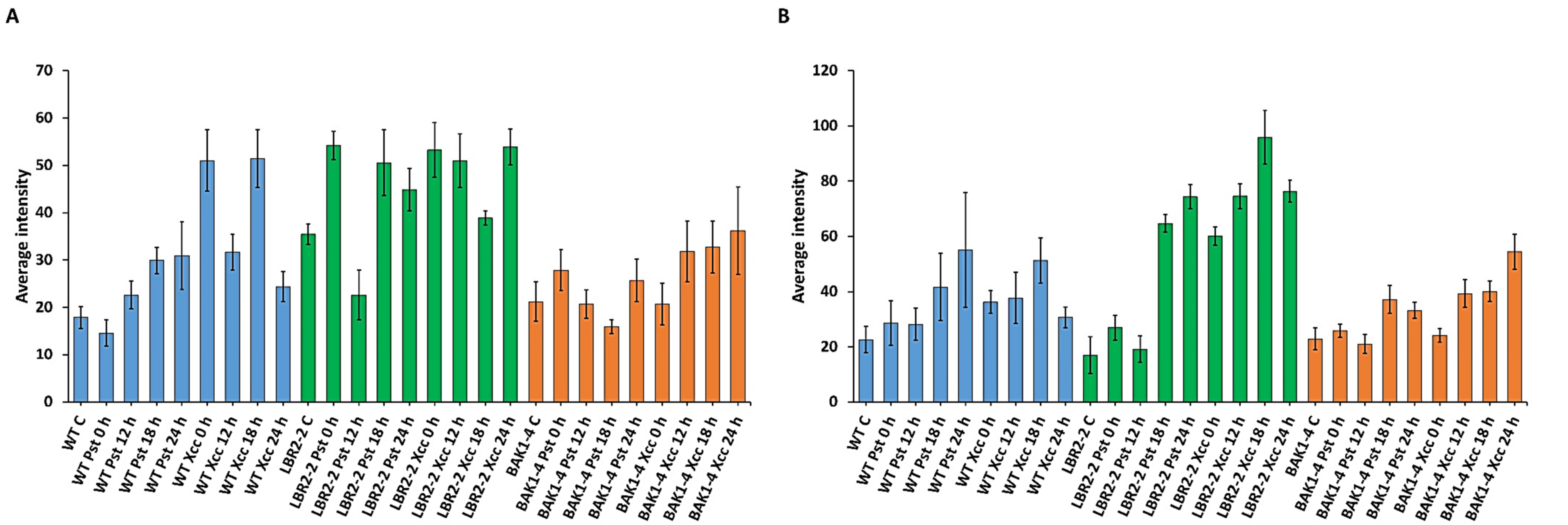

Figure S7. Alpha-linolenic acid metabolism analysis by Metabolomic Pathway Analysis (MetPA). The average intensity of MetPA mapped lipids 13(S)-HPOTrE **(A)** and 12-OPDA **(B)** which represents **#25** and **#32**, respectively, in **Table 1**. Blue, green and orange colour represent Arabidopsis WT, *lbr2-2* and *bak1-4*, respectively.

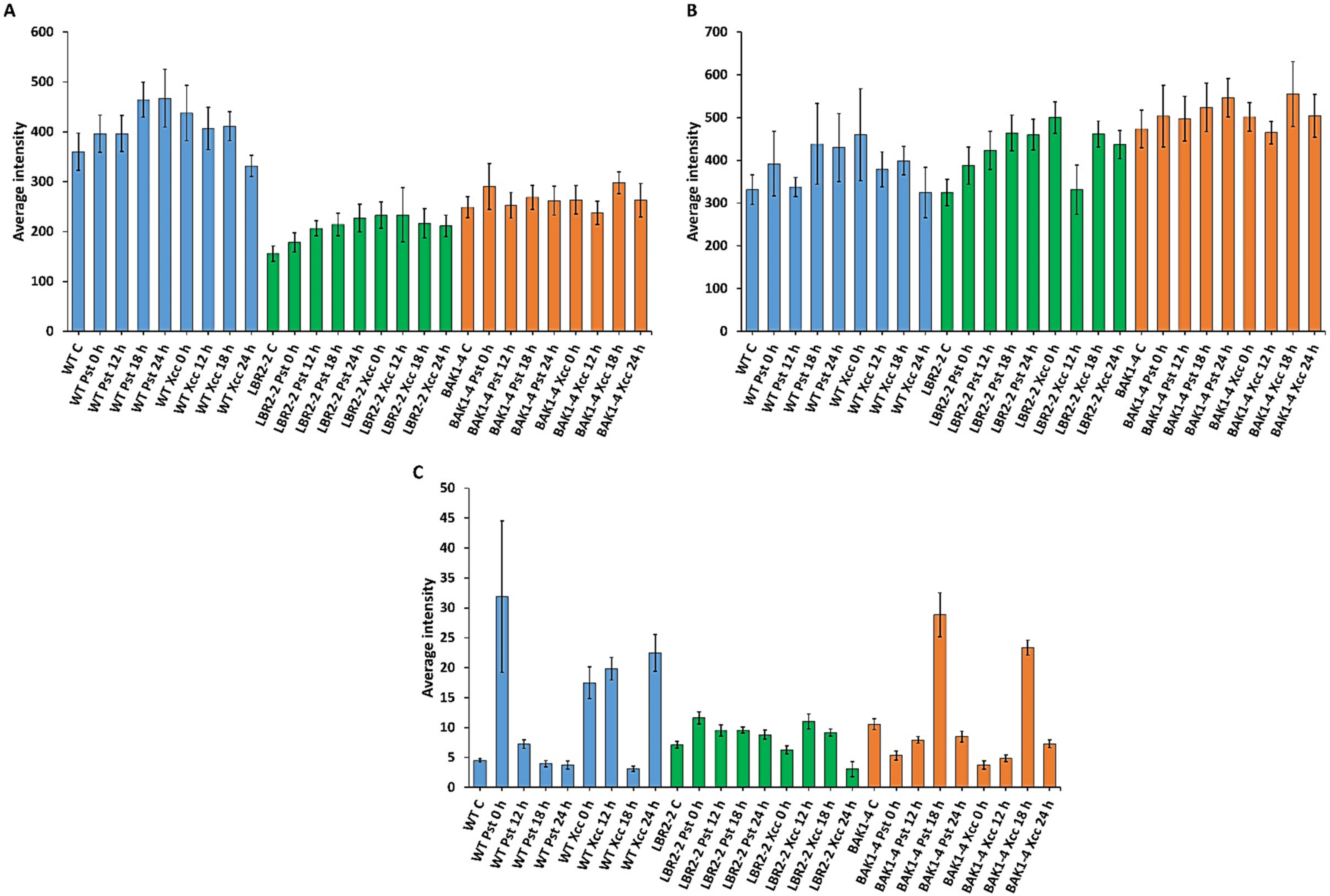

Figure S8. Phenylpropanoid biosynthesis analysis by Metabolomic Pathway Analysis (MetPA). The average intensity of MetPA mapped phenylpropanoid sinapic acid **(A)**, sinapoyl malate **(B)**, and of unmapped phenylpropanoids sinapoyl 1-O-sinapoyl-beta-D-glucose **(C)**, which represents **#9**, **#10** and **#13**, respectively, in **Table 1**. Blue, green and orange colour represent Arabidopsis WT, *lbr2-2* and *bak1-4*, respectively.

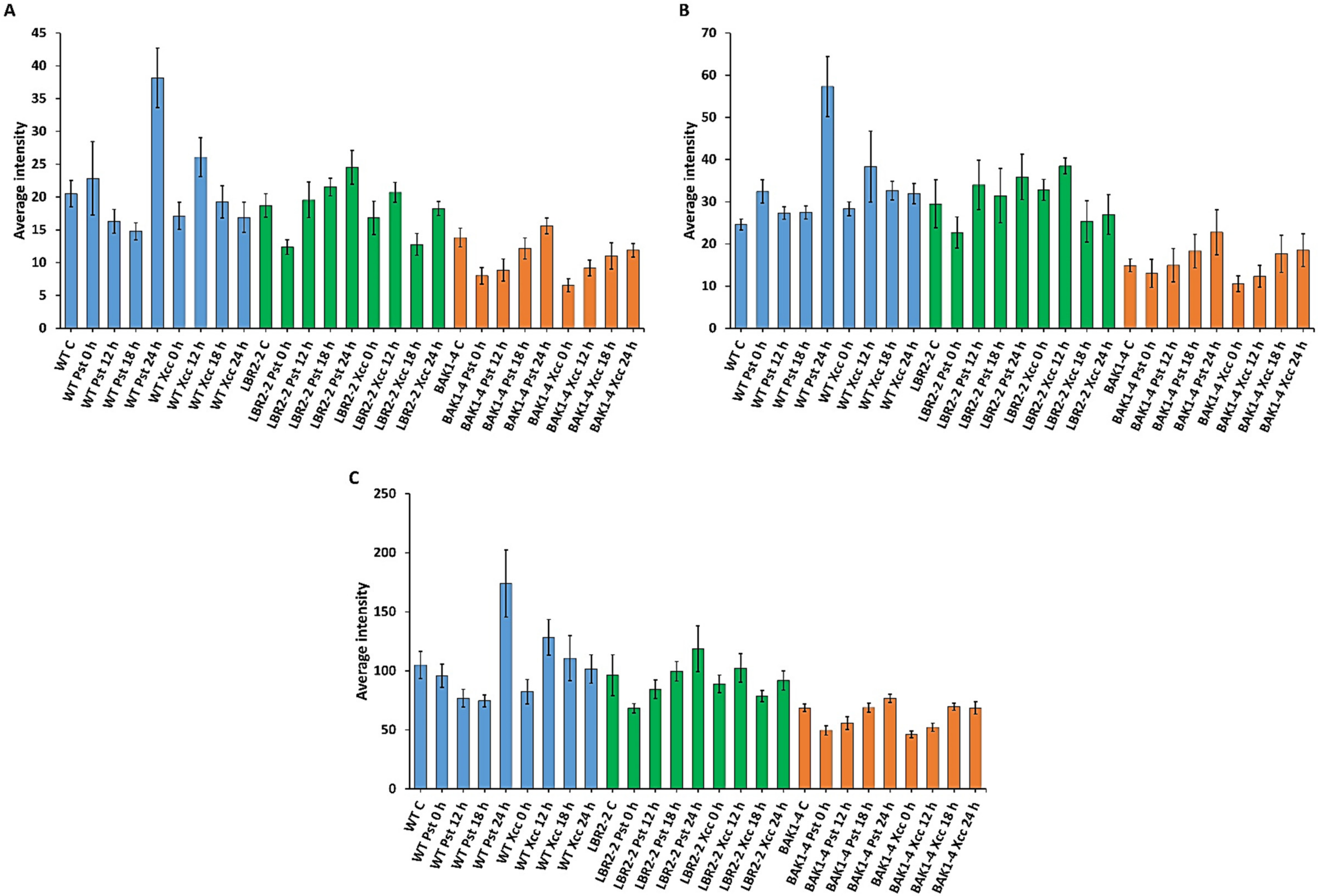

Figure S9. Flavone and flavonol biosynthesis analysis by Metabolomic Pathway Analysis (MetPA). The average intensity of MetPA mapped flavonoids kaempferol-3-O-rhamnoside-7-O-glucoside **(A)** and unmapped flavonoids afzelin (kaempferol-3-rhamnoside) **(B)** and kaempferitrin **(C)**, a which represents **#17**, **#14** and **#16**, respectively, in **Table 1**. Blue, green and orange colour represent Arabidopsis WT, *lbr2-2* and *bak1-4*, respectively.

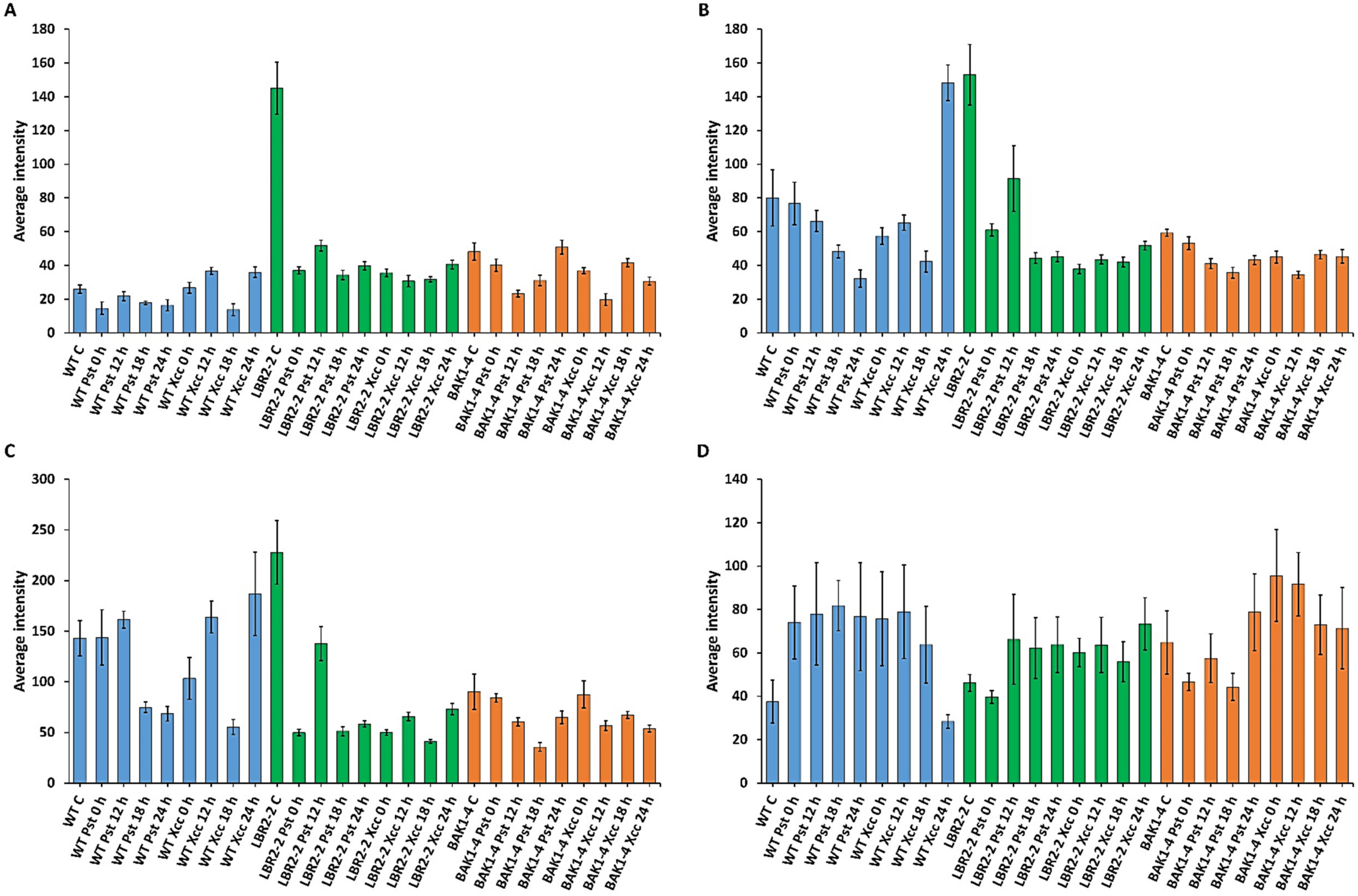

Figure S10. Glucosinolate biosynthesis analysis by Metabolomic Pathway Analysis (MetPA). The average intensity of MetPA mapped glucosinolates glucoerucin **(A)** and glucobrassicin **(B)**, and unmapped glucosinolates glucohirsutin **(C)** and glucosinolate product 8-(methylsulphinyl)octylamine **(D)**, which represents **#5**, **#6**, **#7** and **#4**, respectively, in **Table 1**. Blue, green and orange colour represent Arabidopsis WT, *lbr2-2* and *bak1-4*, respectively.
